# Interventions targeting conscious determinants of human behaviour to reduce the demand for meat: a systematic review with qualitative comparative analysis

**DOI:** 10.1186/s12966-018-0729-6

**Published:** 2018-10-19

**Authors:** Filippo Bianchi, Claudia Dorsel, Emma Garnett, Paul Aveyard, Susan A Jebb

**Affiliations:** 1 Nuffield Department of Primary Care Health Sciences, University of Oxford, Radcliffe Observatory Quarter, Radcliffe Primary Care Building, Woodstock Rd, Oxford, OX2 6GG UK; 2 Department of Psychology, Heinrich Heine University Düsseldorf, Universitätsstraße 1, 40225 Düsseldorf, Germany; 3 Department of Zoology, University of Cambridge, David Attenborough Building, Pembroke Street, Cambridge, CB2 3QZ UK

**Keywords:** Meat consumption, Dietary change, Behaviour change, Interventions, Education, Motivation, Systematic review

## Abstract

**Background:**

Reducing meat consumption can help prevent non-communicable diseases and protect the environment. Interventions targeting conscious determinants of human behaviour are generally acceptable approaches to promote dietary change, but little is known about their effectiveness to reduce the demand for meat.

**Objective:**

To evaluate the effectiveness of interventions targeting conscious determinants of human behaviour to reduce the demand for meat.

**Methods:**

We searched six electronic databases on the 31st of August 2017 with a predefined algorithm, screened publicly accessible resources, contacted authors, and conducted forward and backward reference searches. Eligible studies employed experimental designs to evaluate interventions targeting conscious determinants of human behaviour to reduce the consumption, purchase, or selection of meat in comparison to a control condition, a baseline period, or relative to other eligible interventions. We synthesised results narratively and conducted an exploratory crisp-set Qualitative Comparative Analysis to identify combinations of intervention characteristics associated with significant reductions in the demand for meat.

**Results:**

We included 24 papers reporting on 59 interventions and 25,477 observations. Self-monitoring interventions and individual lifestyle counselling led to, or were associated with reduced meat consumption. Providing information about the health or environmental consequences of eating meat was associated with reduced intentions to consume and select meat in virtual environments, but there was no evidence to suggest this approach influenced actual behaviour. Education about the animal welfare consequences of eating meat was associated with reduced intentions to consume meat, while interventions implicitly highlighting animal suffering were not. Education on multiple consequences of eating meat led to mixed results. Tailored education was not found to reduce actual or intended meat consumption, though few studies assessed this approach.

**Conclusion:**

Some interventions targeting conscious determinants of human behaviour have the potential to reduce the demand for meat. In particular, self-monitoring interventions and individual lifestyle counselling can help to reduce meat consumption. There was evidence of effectiveness of some educational messages in reducing intended consumption and selection of meat in virtual environments.

**Protocol registration:**

CRD42017076720.

**Electronic supplementary material:**

The online version of this article (10.1186/s12966-018-0729-6) contains supplementary material, which is available to authorized users.

## Background

Red and processed meat consumption is associated with an increased risk of developing some non-communicable chronic conditions, including cardiovascular disease [1–3], type-2-diabetes [3–5], and some forms of cancer [6–8]. Additionally, livestock negatively affects the natural environment and advances anthropogenic climate change [9–11]. These environmental changes might in turn affect human health by contributing – among other things – to the pollution of air and drinking water, the rise in antimicrobial resistance, and the spread of vector-borne diseases [12–14]. While the potential health and environmental benefits of reducing meat consumption are well established, concerns about a consumer backlash and the poor understanding of how to promote this behaviour change have contributed to a general state of inaction [15–17]. Interventions targeting conscious determinants of human behaviour, such as those providing information, are generally perceived to be acceptable approaches to promote health behaviours by the public in developed countries [18, 19] and might therefore help to overcome this state of inaction. Furthermore, interventions targeting conscious determinants of human behaviour might, over time, enhance the public’s support for more structural interventions aiming at reducing the demand for meat [15, 16, 20]. Accordingly, identifying effective interventions targeting conscious determinants of human behaviour to reduce the demand for meat is an important step towards promoting healthier and more environmentally sustainable diets. The aim of this systematic review is to synthesise the evidence from studies evaluating the effectiveness of interventions targeting conscious determinants of human behaviour to reduce the actual or intended consumption, purchase, and selection of meat and to identify combinations of intervention characteristics that effectively promote this behaviour change.

## Methods

### Protocol registration and eligibility criteria

A protocol for this systematic review was published on PROSPERO [21]. This review includes studies evaluating interventions targeting conscious determinants of human behaviour to reduce the consumption, purchase, or selection of meat, and that fulfilled the eligibility criteria outlined in Table 1.

**Table 1:** Eligibility Criteria

	Inclusion criteria	Exclusion criteria
Population	All are eligible except those listed in the exclusion criteria.	People diagnosed with clinical condition(s) for which it is required to consume specific amounts of meat.
Intervention	Interventions targeting conscious determinants of human behaviour to reduce the demand for meat, including information provision, motivational interviewing, and interventions aiding self-regulatory processes.	Dietary interventions aiming to promote a general dietary pattern (e.g. interventions promoting the Mediterranean dietary pattern) and interventions restructuring elements of the physical microenvironment (e.g. pricing, positioning, or portion size).
Comparator	In order of preference (1) no- or minimal-intervention controls, (2) pre-intervention baseline, or (3) other eligible intervention(s).	Interventions not fulfilling the eligibility criteria.
Outcome	Objective or self-reported measures of demand for meat, defined as actual or intended consumption, purchase, or selection of meat in real or virtual environments. We extracted data pertaining to the follow-up closest to the intervention completion and to the longest available follow-up, with the former representing our primary outcome.	N/A

We extracted data on participants’ demand for meat, defined as the actual or intended consumption, purchase, or selection of meat. We refer to meat purchases when the selection of meat involves a real or virtual monetary transaction, while we refer to meat selection when no form of monetary transaction is involved. Where reported, we also extracted data pertaining to attitudes, subjective social norms, and perceived behavioural control of eating, purchasing, or selecting meat, and on body weight, blood pressure, blood glucose, and blood lipids. When an outcome was assessed in multiple ways we only extracted data for the most granular measure (e.g. food diary > self-reported score of change) referring to the longest time-span (e.g. consumption over a month > consumption over a week). There were no exclusion criteria pertaining to the length of follow-up, publication status, publication year, or language.

### Search strategy and data extraction

We searched six electronic databases on the 31*st* of August 2017 using a pre-specified search algorithm (Additional file 1: Table S1). We searched for further eligible records contacting researchers and experts, manually conducting forward and backward reference searches, and screening publicly accessible online resources following the methodology described by Stansfield et al. ( [22], Additional file 1: Table S2). Two members of the research team independently assessed the eligibility of the studies, extracted data from eligible records using a pre-piloted data extraction form, and evaluated the methodological quality of all eligible studies using the Quality Assessment Tool for Quantitative Studies [23]. Where additional information was required, we contacted authors and/or reviewed study protocols. Disagreements were resolved by discussion and by referral to a third member of the research team.

### Data synthesis

We synthesised results narratively grouping interventions in five categories: (1) individual lifestyle counselling, (2) goal-setting and self-monitoring interventions, (3) non-tailored education (about meat consumption and one or more of health, the environment, animal welfare, socio-economic issues), (4) tailored education, and (5) interventions implicitly highlighting animal suffering. We augmented our narrative synthesis with an exploratory crisp-set Qualitative Comparative Analysis (QCA). In our QCA we used a binary coding system to categorise each intervention as presenting or not presenting specific behavioural strategies or implementation features to identify configurations of intervention characteristics associated with (and those not associated with) statistically significant (*p* < 0.05) reductions in the demand for meat at the follow-up closest to the intervention completion. Where studies reported results for multiple eligible outcomes we only considered the outcome closest to ‘actual behaviour’ (actual behaviour > behaviour in virtual environments > intention). The intervention characteristics included in QCA pertained to whether an intervention provided information about the (a) health, (b) environmental, (c) animal welfare, (d) socio-economic, (e) and multiple consequences of meat consumption, and whether it (e) provided tailored information, (f) employed self-monitoring strategies, (g) employed goal-setting strategies, (h) implicitly highlighted animal suffering, (i) implemented individual lifestyle counselling sessions, (j) targeted people with, or at increased risk of ill-health (k) provided information on ‘how to’ eat less meat, and whether (l) the outcome was actual as opposed to intentions or behaviour in virtual environments. In QCA we included comparisons between interventions and no-intervention controls or pre-intervention baselines, while comparisons between multiple interventions were excluded. A more detailed account on QCA in systematic reviews is outlined by Thomas et al. [24].

## Results

### Study selection

**Fig. 1:**
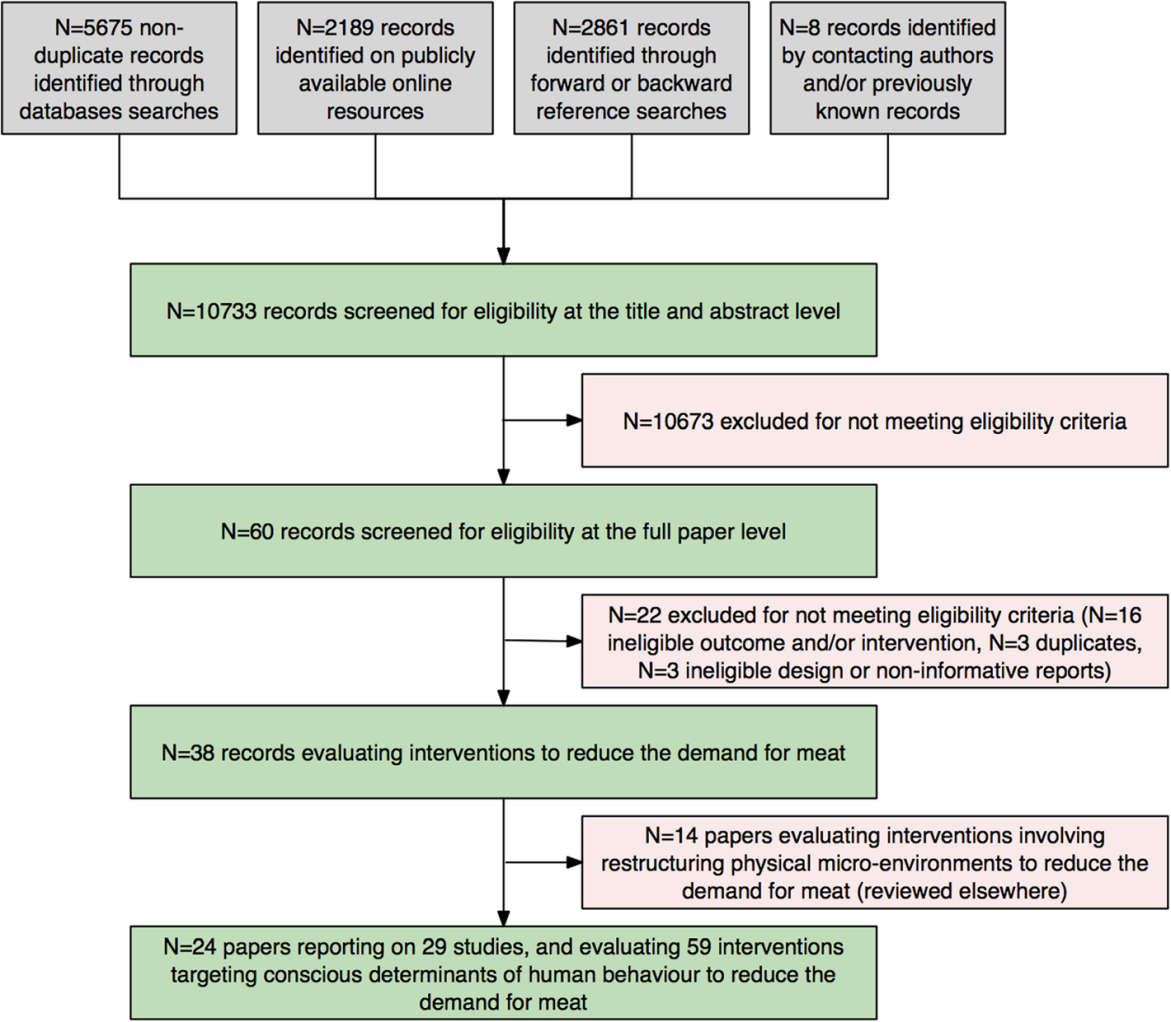
Study flow diagram

We screened 10,733 titles/abstracts, assessed the full-text of 60 papers, and included 24 papers reporting on 29 studies and 59 intervention conditions (Fig. 1). The majority of papers (54%) were published or written between 2015 and 2017.

### Study characteristics

This review includes 25,477 observations (i.e. individuals or individual meal purchases) at the follow-up closest to the intervention completion and 305 observations at the longest follow-up for the four studies reporting on multiple end-points. Where reported, mean age ranged from 19 to 74 years (median = 36), the proportion of female participants ranged from 49 to 100% (median = 61%), the proportion of white participants ranged from 58 to 83% (median = 75%), and participants’ education and/or household income were high in most studies. Forty-five percent of studies only included individuals who ate meat. Studies recruited individuals (*N* = 26) or food providers (*N* = 3) through active recruitment (*N* = 15), advertisement and passive recruitment (*N* = 8), or through online panels (*N* = 6). Studies were conducted in Europe (*N* = 12), the USA (*N* = 9), China (N = 2), New Zealand (N = 2), and Brazil (N = 1), and were implemented in various settings, including experimental settings (*N* = 18), canteens or food services (N = 3), healthcare settings (N = 2), and among free-living individuals (N = 6). Fourteen studies employed a randomised controlled trial (RCT), 5 employed a non-randomised controlled trial (CT), 1 study employed a crossover design, 7 employed a pre-post design, and 2 studies retrospectively evaluated the intervention effectiveness. Retention at the shortest follow-up ranged from 59 to 100% (median = 95%). The overall methodological quality was ‘strong’ for 6 studies, ‘medium’ for 10 studies, and ‘weak’ for 13 studies. Table 2 summarises the characteristics of all studies included.

**Table 2:** Study-level characteristics by study design underlying the main comparison reported in this review

Reference and country	Eligibility criteria	Recruitment strategy	Attrition and sample size (a, b)	Availability	*EPHPP QATQS* score (c)
Randomised Controlled Trials (RCT)					
Arndt, 2016, study 1, USA [43]	Exclusion: Individuals who seldom or never ate meat, did not eat meat weekly, consumed no meat containing meal in the past 3 days, ate no serving of meat on an average day, less than 10% of what they ate on an average day is meat, believed that eating meat is bad, disliked eating meat, identified as vegetarians or vegans.	Individual recruitment through Amazon Mechanical Turk.	T1: 0% (179 to 179)	Unpublished, available online	Low
Arndt, 2016, study 2, USA [43]	See Arndt 2016, study 1.	See Arndt 2016, study 1.	T1: 0% (296 to 296)	Unpublished, available online	Low
Carfora et al., 2017, Italy [32]	Exclusion: Individuals following specific diets (such as vegan, vegetarian, protein, slimming and/or fattening diets).	E-mails were sent to a convenience sample of Italian undergraduates.	T1: 9.68% (124 to112)	Peer reviewed publication	Medium
Carfora et al., 2017, Italy [31]	Participants had to have a mobile phone supporting SMS. Exclusion: Individuals following specific diets or who participated to the other study by Carfora.	See Carfora et al., 2017 above.	T1: 4.2% (238 to 228)	Peer reviewed publication	Medium
Emmons et al., 2005, USA [25]	Participants had to be between 40 and 75 years, have an adenomatous colon polyp removed within 4 weeks of recruitment, have no personal history of CRC, be competent in English, be capable of informed consent, and be reachable by phone.	Eligible individuals were sent a letter describing the study and were later contacted by phone unless they opted out.	T1: 12.59% (1247 to 1090)	Peer reviewed publication	Strong
Emmons et al., 2005, USA [26]	Participants had to be 18 to 75 years old, have a visit scheduled with a participating healthcare provider, be competent in English or Spanish, and come from an eligible working-class neighbourhood. Exclusion: Individuals who had cancer at enrolment, or who were employed by the participating health centres or at a worksite participating in the companion study (Emmons et al., 2005 (a)).	See Emmons et al., 2005 above.	T1: 12% (2219 to 1954)	Peer reviewed publication	Medium
Fehrenbach, 2013, USA [33]	Exclusion: Individuals who were vegan, vegetarian, or pescetarian, self-reported insufficient attention to the message, previously completed part or all of the survey, had incomplete data, received the wrong intervention, and international or non-undergraduate students.	Individuals were sampled from communication classes at a large university in Arizona in exchange for extra credits.	T1: 10.1% (208 to 187)	Unpublished, not available online	Low
Fehrenbach, 2015, USA [37]	Participants had to be U.S. resident, 25–44 years of age and consume meat 7+ times/week. Exclusion: Individuals who took the survey on mobile devices, failed an attention filter, or completed the survey too quickly or without viewing the video.	Individual recruitment from a national panel using Qualtrics.	T1: 1.61% (373 to 367)	Unpublished, available online	Low
			T2: 58.98% (373 to 153)		
Graham et al., 2017, New Zealand [39]	Participants had to reside in New Zealand and pass an attention filter.	Individual recruitment through convenience and snowball techniques on a university campus, and advertisement outside the university campus.	T1: 0%	Peer reviewed publication	Medium
Klöckner et al., 2017, study 1, Netherlands [44]	Participants had to be adult Norwegians.	Individuals were randomly selected from the population registry and sent an invitation letter.	T1: 17.1% (1047 to 868)	Peer reviewed publication	Low
Klöckner et al., 2017, study 2, Netherlands [44]	Participants had to be adults.	Individuals were recruited from the professional online panel TNS Gallup.	T1: 8.63% (3895 to 3559)	Peer reviewed publication	Medium
Tian et al., 2016, study 1, France and China [49]	Exclusion: Individuals who identified as vegetarians.	Individuals were recruited using social media and internal university advertisement.	T1: 41.47% (885 to 518)	Peer reviewed publication	Low
Tian et al., 2016, study 2, France and China [49]	See Tian et al., 2016, study 1.	See Tian et al., 2016, study 1.	T1: 14.52% (606 to 518)	Peer reviewed publication	Low
Vibhuti, 2016, USA [40]	Participants had to be adults and reside in the US.	Individual recruitment through Amazon Mechanical Turk.	T1: 0,97% (412 to 408)	Unpublished, available online	Low
Non-randomised Controlled Trials (CT)					
Allen et al., 2012, Australia [42]	N/A	The survey was sent to a random sample of individuals drawn from the telephone directory.	T1: 1.82% (220 to 216)	Peer reviewed publication	Low
			T2: 55.91% (220 to 97)		
Berndsen et al., 2005, study 1, Netherlands [34]	Participants had to be meat eaters.	Individual recruitment through internal university advertisement	T1: 0% (141 to 141)	Peer reviewed publication	Low
			T2: 0% (141 to 141)		
Berndsen et al., 2005, study 2, Netherlands [34]	See Berndsen et al., 2005, study 1.	See Berndsen et al., 2005, study 1.	T1: 0% (92 to 92)	Peer reviewed publication	Low
			T2: 0% (92 to 92)		
Bertolotti et al., 2016, Italy [38]	Participants had to be over 60 years old, had to volunteer to participate, and complete sufficient sections of the questionnaire.	Active recruitment of individuals from socio-recreational centres for the elderly in Milan, Italy.	T1: 19.17% (120 to 97)	Peer reviewed publication	Strong
Schiavon et al., 2015, Brazil [27]	All patients admitted for surgical treatment of suspected malignant breast tumors in the Maternidade Carmela Dutra Hospital. Exclusion: Individuals who had a history of cancer or a surgical procedure in the previous year; were pregnant or breastfeeding at the time of diagnosis; had positive results for HIV; had neoadjuvant cancer treatment, or a neurological disease.	Active recruitment of all aforementioned patients.	T1: 9.71% (103 to 93)	Peer reviewed publication	Strong
Crossover design (CO)					
Scrimgeour, 2012, New Zealand [35]	N/A	Individuals were recruited using the University Psychology and Geography mailing lists and snowballing techniques	T1: 18.66% (434 to 353)	Unpublished, available online	Medium
Single group pre-post design					
Cordts et al., 2014, Germany [36]	Participants had to be meat eaters.	Individual recruitment through a professional panel provider with the aim of obtaining a representative sample of the German population.	T1: 5.76% (590 to 556)	Peer reviewed publication	Strong
Godfrey, 2014, Canada [41]	N/A	Food stations were recruited from the University Dining Centre at the University of Calgary.	T1: N/A (16,786 meal purchases)	Unpublished, available online	Medium
Grimmet et al., 2016, UK [30]	Participants had to be over 18 years, have completed treatment for non-metastatic CRC within the last 6 months, be competent in English, have adequate mobility and no contraindications for unsupervised physical activity.	Consultants in 3 London hospitals referred patients to the researchers and research-nurses recruited participants from 5 London hospitals.	T1: 20.69% (29 to 23)	Peer reviewed publication	Medium
Hawkes et al., 2009, Australia [28]	Participants had to be 20–80 years old, approximately 6 months post-CRC diagnosis; competent in English; and have no hearing, speech, or cognitive disabilities preventing them from completing telephone interviews.	Invitation and consent packages were sent to individuals who had undergone treatment in 3 the practices of three practitioners in Brisbane.	T1: 0% (20 to 20)	Peer reviewed publication	Strong
Hawkes et al., 2012, Australia [29]	Participants had to be able to understand and give informed consent in English; have no current or previous diagnosis of CRC or medical conditions limiting adherence to an unsupervised lifestyle program; own a phone; and have one or more poor health behaviour(s) among: not achieving ≥150 min of physical activity/week; eating > 4 servings of red meat/week or < 2 serves of fruit/day, or < 5 servings of vegetables/day; consuming > 2 drinks/day; or if they had a BMI ≥25. Participants had to have a first degree relative with CRC.	Social media, printed material, radio and online advertisement.	T1: 0% (22 to 22)	Peer reviewed publication	Medium
Loy et al., 2016, Germany [45]	Participants had to be non-vegetarians and proficient in German.	Individuals were recruited through internal university advertisement.	T1: 3,33% (60 to 58)	Peer reviewed publication	Medium
			T2: 8.33% (60 to 55)		
Marette et al., 2016, France [46]	Participants had to eat ground beef, at least occasionally.	Individuals were recruited via phone to randomly select a sample representative of the age groups and socio-economic status of the population in Dijon, France.	T1: 3,23% (124 to 120)	Report, available online	Strong
Retrospective intervention evaluation					
Leidig, 2012, study 1, USA [47]	All healthcare accounts of Sodexo’s food service in the USA were eligible.	The survey was distributed to the managers of all USA healthcare accounts.	T1: N/A (119 account managers)	Report, available online	Low
Leidig, 2012, study 2, USA [47]	All corporate and governmental accounts of Sodexo’s food service in the USA were eligible.	The survey was distributed to the managers of all USA corporate and government accounts.	T1: N/A (126 account managers)	Report, available online	Low

Legend: (a) T1 and T2 respectively refer to the follow up closest to the intervention completion and to the longest available follow-up. (b) Studies reporting no attrition may have reported data of completers only throughout the paper. (c) The *EPHPP QATQS* (Effective Public Health Practice Project Quality Assessment Tool For Quantitative Studies) score indicates the study’s methodological quality and is based on: (1) study design, (2) selection bias, (3) confounders, (4) blinding, (5) data collection method, (6) withdrawal and dropouts. Studies with ≥2 weak ratings in the aforementioned dimensions are assigned a ‘low’ score, studies with 1 weak rating are assigned a ‘medium’ score, and studies with no weak rating are assigned a ‘strong’ score

### Interventions and outcomes

We included 37 interventions providing non-tailored information about meat consumption and health (*N* = 11), the environment (*N* = 8), animal welfare (*N* = 2), socio-economic issues (N = 2), or a combination thereof (*N* = 14). Ten interventions provided tailored education, four implicitly highlighted animal suffering, six delivered individual lifestyle counselling, and two delivered self-monitoring interventions. Of all interventions included, two interventions targeted food suppliers while the rest targeted consumers. Fifteen studies reported on participants’ actual meat consumption, 6 on meat purchase or selection, and 15 on intended consumption. Fifteen studies additionally reported on other pre-specified secondary psychosocial outcomes or biomarkers of health risk. Table 3 summarises each intervention and its impact on, or association with participants’ demand for meat at the shortest follow-up. The results for the longest follow-up and for other secondary psychosocial and biological outcomes are summarized in Additional file 1: Tables S3, S4, and S5.

**Table 3:** Interventions’ impact on or association with the demand for meat at the shortest follow-up

Paper	Sample characteristics and comparison (a)	Intervention	Outcome	Results (b)	Direction of outcome
Individual lifestyle counselling					
Emmons et al., 2005, [26].	Sample size: IG: *N* = 1088, CG: *N* = 1131 Age: M = 49 Female: 66% Comparison: IG vs CG, RCT	The intervention targeted consumption of red meat, fruit and vegetable, multivitamin, and physical activity. It comprised endorsement and tailored prescription to prompt behavior change by participants’ clinician, 1 × 20′ in-person and 4 × 10′ telephone counseling sessions with a health advisor, and tailored supporting material including information on barriers to change. The intervention focused on social determinants of behavior. CG: Usual care.	The proportion of participants reporting consuming ≤3 servings/w of red meat over the past 4 weeks was assessed at the baseline and 8 months later with a semi-quantitative Food Frequency Questionnaire (FFQ).	In the IG the proportion of participants consuming ≤3 servings/w rose by 11.8%, while in the CG it decreased by 0.2%. The changes over time between the two conditions were significantly different (*p* < 0.001).	Desired direction
Emmons et al. (a), 2005, [25].	Sample size: IG: *N* = 591, CG: *N* = 656 Age: Median: within 60–75 Female: 42% Comparison: IG vs CG, RCT	IG: The intervention targeted consumption of red meat, fruit and vegetable, alcohol, multivitamin, physical activity, and smoking. It comprised 1 motivational and goal-setting telephone session and 4 telephone counselling sessions delivered at monthly intervals by health advisors, and tailored supporting materials. CG: Usual care, gastroenterologist endorsement of the study’s behavioural targets, and CRC prevention leaflet.	The proportion of participants reporting consuming on average ≤ 3 portions/w of red meat was assessed at the baseline and 8 months later with a semi-quantitative FFQ.	Compared to the CG (12%) more participants in the IG reduced their meat intakes to < 3 portions/w (18%, *p* = 0.002).	Desired direction
Schiavon et al., 2015, [27].	Sample size: IG: *N* = 18, CG: *N* = 75 Age: M = 51 Female: 100% Comparison: IG vs CG, CT	IG: The 12 month intervention targeted consumption of red and processed meat and fruit and vegetables. It provided information bi-weekly phone calls, bimonthly 24-h dietary recalls followed by researchers’ feedback, and supporting material. CG: Basic healthy lifestyle guidelines at the baseline and follow-up.	Red and processed meat consumption (in g/d) was assessed with an FFQ for Brazilian diets, directly post-intervention.	There was a significant difference in red and processed meat consumption between the groups in unadjusted analyses (B(exp) = 0.5, *p* < 0.05) and in analyses adjusting for post-intervention energy intake and baseline red and processed meat consumption (B(exp) = 0.6, *p* < 0.05). This effect was not detected when also adjusting for baseline saturated and monounsaturated fat, and carbonyl protein and reduced glutathione (B(exp) = 0.6, *p* > 0.05).	Desired direction
Grimmet et al., 2016, [30].	Sample size: *N* = 29 Age: M = 65 Female: 62% Comparison: Pre-post	IG: The 12 week intervention targeted consumption of red and processed meat, fruit and vegetables, and physical activity. It comprised 2 weekly telephone calls from the researcher and supporting materials including recipes. The intervention focused on goal setting, review of goals, self-monitoring, and feedback on performance.	Consumption of red (in g/w) and processed meat (in portions/w) was assessed with an FFQ before and directly post-intervention.	Red and processed meat consumption decreased from pre- to post-intervention (mean reduction for red meat: 147.4, *p* = 0.013; mean reduction for processed meat: 0.83, *p* = 0.002).	Desired direction
Hawkes et al., 2009, [28].	Sample size: *N* = 20 Age: Median: 66 Female: 50% Comparison: Pre-post	IG: The 6 week intervention targeted consumption of red and processed meat, fruit and vegetable, alcohol, weight management, physical activity, and smoking. It comprised 6 weekly 45′ telephone counselling sessions from a trained health coach, and supporting material. The intervention included lifestyle support, health risks information, behaviour change strategies, self-efficacy, and outcome expectations.	Consumption of red and processed meat (in servings/w) was assessed via phone, before and directly post-intervention.	There was a significant decrease in the intake of processed meat servings/w from baseline (Median = 1) to post intervention (Median = 0, *p* = 0.01). The proportion of participants eating ≤3–4 servings/w of red meat did not change from pre- (85%) to post-intervention (85%, *p* = 1).	Desired direction (processed meat) No association (red meat)
Hawkes et al., 2012, [29].	Sample size: *N* = 22 Age: M = 47 Female: 82% Comparison: Pre-post	IG: The 6 week intervention targeted consumption of red and processed meat, fruit and vegetable, alcohol, weight management, physical activity, and smoking. It comprised 6 × 1-h telephone-coaching sessions with a trained health coach, focussing on motivation, expectations, values, mindfulness, action planning, goal-setting, and self-monitoring, and supporting material.	Consumption of red and processed meat (in servings/w) was assessed via phone before and directly post-intervention.	Processed meat consumption declined from pre- to post-intervention (mean change, 95%CI = − 1.2, − 1.8 to − 0.5, *p* < 0.01). Red meat consumption did not change from pre- to post-intervention (mean change, 95%CI = 0.02, − 0.6 to 0.6, *p* = 0.93).	Desired direction (processed meat) Undesired direction (red meat)
Self-monitoring and goal setting interventions					
Carfora et al., 2017, [32],	Sample size: IG: *N* = 57, CG: *N* = 55 Age: M = 19 Female: 56% Comparison: IG vs CG, RCT	IG: Daily text messages for a week, encouraging participants to self-monitor their consumption of processed meat and to ‘think about the regret they could experience’ if they were to exceed the recommended levels of processed meat consumption (50 g/d). CG: No intervention.	Consumption of processed meat (in servings) was assessed using a 7-day food diary during the week preceding and the week concomitant to the intervention. Intention to eat ≤50 g of processed meat over the upcoming week was assessed with three items on a scale from 1 (strongly disagree) to 7 (strongly agree) before and directly post-intervention.	During the intervention the IG ate significantly fewer servings of processed meat (M = 1.74) than the CG (M = 3.29, *p* < 0.001, d = 0.7). At post- intervention, the IG reported higher intentions to eat ≤1 serving of processed meat in the upcoming week (M = 4.47) compared to the CG (M = 3.60, *p* < 0.008, d = 0.51).	Desired direction
Carfora et al., 2017(a), [31].	Sample size: IG: *N* = 116, CG: *N* = 112 Age: M = 19 Female: 72% Comparison: IG vs CG, RCT	IG: Daily text messages for a week, encouraging participants to self-monitor their consumption of red meat to not exceed a recommended maximum of two medium servings per week. CG: No intervention.	Consumption of red meat (in servings) was assessed using a 7-day food diary during the week preceding and the week concomitant to the intervention. Intention to eat < 2 portions of red meat over the upcoming week was assessed with three items on a scale from 1 (strongly disagree) to 7 (strongly agree) before and directly post-intervention.	During the intervention the IG ate significantly fewer servings of red meat (M = 1.62) than the CG (M = 3.03, *p* < 0.001, d = 0.74). At post- intervention, the IG reported higher intentions to eat < 2 servings of red meat over the upcoming week (M = 4.80) compared to the CG (M = 4.07, *p* < 0.01, d = 0.41).	Desired direction
Non-tailored information about meat consumption and health					
Fehren-bach, 2013, [33].	Sample size: *N* = 187 (total study) Age: Median: within 18–25 (total study) Female: 57% (total study) Comparison: IG vs CG, RCT	IG: A webpage on the health impact of eating meat, recommending practical strategies to eat less meat. CG: A webpage about the Rolling Stones.	Intention to reduce meat consumption was measured directly post-intervention, with three items on a scale from 1 (strongly disagree) to 5 (strongly agree).	Post-intervention intention to eat less meat was higher in the IG (M = 3.90) than in the CG (M = 2.69, *p* < 0.001).	Desired direction
Fehren-bach, 2015, [37].	Sample size: IG: *N* = 124, CG: N = 124 Age: 57 (total study) Female: 57 (total study) Comparison: IG vs CG, RCT	IG: A 4′ video about the health impact of eating meat, highlighting participants’ susceptibility to these outcomes. CG: No intervention.	At the baseline and one week post-intervention, participants reported how many meat-containing meals they ate in the past 7 days. Intention to eat less meat in the upcoming 7 days was assessed with three 5-points scales, directly post-intervention.	Meat intakes did not differ between the IG (M = − 3.16) and the CG (M = − 1.92) or any other study groups (*p* = 0.31, d = 0.29). Intention to eat less meat was higher in the IG (M = 3.46) than in the CG (M = 2.57, *p* < 0.001).	Desired direction
Fehren-bach, 2015, [37].	Sample size: IG: N = 124, CG: N = 124 Age: 57 (total study) Female: 57 (total study) Comparison: IG vs CG, RCT	IG: A 7′ video about the negative health outcomes of eating meat, highlighting participants’ susceptibility to these outcomes, the health benefits of low meat diets, and strategies to eat less meat. CG: No intervention.	At the baseline and one week post-intervention, participants reported how many meat-containing meals they ate in the past 7 days. Intention to eat less meat in the upcoming 7 days was measured with three 5-points scales, directly post-intervention.	Meat intakes did not differ between the IG (M = − 2.11) and the CG (M = − 1.92), or any other study groups (*p* = 0.31, d *=* 0.29). Intention to eat less meat was higher in the IG (M = 3.69) than in the CG (M = 2.57, *p* < 0.001).	Desired direction
Berndsen et al., 2005, study 1, [34].	Sample size: IG: *N* = 50, CG: *N* = 38 Age: M = 20 (total study) Female: 59% (total study) Comparison: IG vs. CG, CT	IG: Cognitively framed paragraph on the health consequences of eating meat. CG: No intervention.	Three weeks post-intervention, participants reported whether they ate less meat in the past 3 weeks. Directly post-intervention, participants reported if they intended to eat less meat over the upcoming 3 weeks. The scales ranged from 1 (fully disagree) to 9 (fully agree).	Self-reported change in meat consumption did not differ between the IG (M = 2.78) and CG (M = 3.16, *p* > 0.05). Intention to eat less meat did not differ between the IG (M = 2.38) and the CG (M = 3.08, *p* > 0.05).	Undesired direction
Scrim-geour, 2012, [35].	Sample size: *N* = 363 Age: M = 28 Female: 68% Comparison: Pre-post, Crossover	IG: Information paragraph on the health impact of eating less meat.	Whether participants intended to eat less, the same, or more meat in the future was assessed pre- and post-intervention.	Compared to the baseline (M ≈ 2.18), participants’ intention to eat less meat was higher after the intervention (M ≈ 2.26, *p* < 0.001, d = 0.59). (c)	Desired direction
Cordts et al., 2014, [36].	Sample size: *N* = 136 Age: Median: within 40–59 (total study) Female: 45% Comparison: Pre-post	IG: Article on the health impact of eating meat.	The number of participants intending to eat less meat was assessed pre- and post-intervention by asking whether they would eat less, the same, or more meat in the future.	The percentage of participants intending to reduce meat consumption increased from pre- (13.1%, *N* = 137) to post-intervention (23.5%, N = 136, *p* < 0.001).	Desired direction
Berndsen et al., 2005, study 1, [34].	Sample size: IG1: *N* = 53, IG2: N = 50 Age: M = 20 (total study) Female: 59% (for total study) Comparison: IG1 vs. IG2, CT	IG1: Affectively framed paragraph on the health impact of eating less meat. IG2: Cognitively framed paragraph on the health impact of eating less meat.	Three weeks post-intervention, participants reported whether they ate less meat in the past 3 weeks. Directly post-intervention, participants reported if they intended to eat less meat over the upcoming 3 weeks. The scales ranged from 1(fully disagree) to 9(fully agree).	Self-reported change in meat consumption did not differ between IG1 (M = 3.75) and IG2 (M = 2.78, *p* = 0.07). Directly post-intervention, IG1 (M = 3.48) had higher intentions to eat less meat over the upcoming 3 weeks than IG2 (M = 2.38, *p* < 0.05). (d)	N/A
Bertolotti et al. 2016, [38].	Sample size: IG1: *N* = 25, IG2: *N* = 23, IG3: *N* = 24, IG4: N = 25 Age: M = 74 (total study) Female: 73% (total study) Comparison: IG1 vs. IG2, IG3 vs. IG4, IG1 vs IG4, CT	IG1: Factually framed paragraph on the health impact of eating less meat. IG2: Pre-factually framed paragraph on the health impact of eating less meat. IG3: Factually framed paragraph on the well-being impact of eating less meat. IG4: Pre-factually framed paragraph on the well-being impact of eating less meat.	Selection of meat dishes was assessed in a simulated food choice task directly post-intervention. Intention to eat red and processed meat in the upcoming month was assessed on a scale from 1 (“much less than before”) to 7 (“much more than before”) at post-intervention.	At post-intervention, IG1 chose fewer meat dishes than IG2 (*p* = 0.015) but had not significantly lower intentions to eat red or processed meat (*p* > 0.07). IG4 did not choose fewer meat dishes than IG3 (*p* = 0.089) but had lower intention to eat red (*p* = 0.046) and processed meat (*p* = 0.035). Intention to eat red and processed meat did not differ significantly between IG1 and IG4 (*p* > 0.28). There was no main effect of content (health vs well-being) or frame (pre-factual vs factual) on any outcome.	N/A
Information about meat and the environment					
Fehren-bach, 2013, [33].	Sample size: *N* = 187 (total study) Age: Median: within 18–25 (total study) Female: 57% (total study) Comparison: IG vs CG, RCT	IG: A webpage on the environmental impact of eating meat, recommending practical strategies to eat less meat. CG: Control web-site on the Rolling Stones.	Intention to reduce meat consumption was measured directly post-intervention, with three items on a scale from 1 (strongly disagree) to 5 (strongly agree).	Intention to eat less meat was higher in the IG (M = 3.71) than in the CG (M = 2.69, *p* < 0.001).	Desired direction
Graham, 2017, [39].	Sample size: IG: *N* = 264, CG: *N* = 317 Age: Median: within 21–30 (total study) Female: 69% (total study) Comparison: IG vs CG, RCT	IG: A self-transcendent framed paragraph on the livestock related GHG emissions in NZ and the mitigation potential of reduced consumption.	Intention to eat meat in the upcoming month was assessed with three items on a scale from 1 (low intention) to 7 (high intention), directly post-intervention.	Intention to eat meat was lower in the IG (M = 3.9) than in the CG (M = 4.2, *p* < 0.05).	Desired direction
Graham, 2017, [39].	Sample size: IG: *N* = 267, CG: N = 317 Age: Median: within 21–30 (total study) Female: 69% (total study) Comparison: IG vs CG, RCT	IG: A self-enhancement framed paragraph on the livestock related GHG emissions in NZ and the mitigation potential of reduced consumption.	Intention to eat meat in the upcoming month was assessed with three items on a scale from 1 (low intention) to 7 (high intention), directly post-intervention.	Intention to eat meat was lower in the IG (M = 4.0) than in the CG (M = 4.2, *p* < 0.05).	Desired direction
Vibhuti, 2016, [40].	Sample size: IG: *N* = 183, CG: *N* = 225 Age: M = 36 Female: 54% Comparison: IG vs CG, RCT	IG: An essay on the environmental impact of the meat consumption and production. CG: No intervention.	Directly post-intervention participants completed six virtual food choices, selecting between a meat-based food and a comparable meat-free alternative.	The proportion of selected meat and meat-free products differed significantly between the CG (meat≈61%) and the IG (meat≈55%, *p* = 0.003, V = 0.06).	Desired direction
Scrim-geour, 2012, [35].	Sample size: N = 363 Age: M = 28 Female: 68% Comparison: Pre-post, Crossover	IG: Information paragraph on the environmental impact of eating meat and strategies to reduce consumption.	Whether participants intended to eat less, the same, or more meat in the future was assessed pre- and post-intervention.	Intention to eat less meat was higher at post-intervention (M ≈ 2.27) than at the baseline (M ≈ 2.18, *p* < 0.001, d = 0.27).	Desired direction
Cordts et al., 2014, [36].	Sample size: N = 128 Age: Median: within 40–59 (total study) Female: 45% Comparison: Pre-post	IG: An article on the environmental impact of eating meat.	The number of participants intending to eat less meat was assessed pre- and post-intervention by asking whether they would eat less, the same, or more meat in the future.	The percentage of participants intending to eat less meat increased from pre- (13.4%, *N* = 127) to post- intervention (18.8%, *N* = 128, *p* < 0.001).	Desired direction
Godfrey, 2014, [41].	Sample size: *N* = 6758 purchases during the intervention period, N = 4426 purchases during the control period. Comparison: Pre-post	IG: A poster on the water footprint of meals containing vegetables (300 L/meal), pork, chicken or fish (590 L/meal), and beef (1350 L/meal) was displayed over 2 weeks in a university canteen.	Meat purchases were assessed using production reports of the dining centres indicating how many servings of each main course were made daily.	There was no difference in the proportion of meat dishes purchased in the control period (87.19%) and in the intervention period (87.82%, *p* > 0.05).	Undesired direction
Godfrey, 2014, [41].	Sample size: *N* = 1176 purchases during the intervention period, *N* = 4426 purchases during the control period. Comparison: Pre-post	IG: A poster on the water footprint of meals containing vegetables (300 L/meal), pork, chicken or fish (590 L/meal), and beef (1350 L/meal) and mentioning the daily water-friendly option was displayed for 4 days in a university canteen.	Meat purchases were assessed using production reports of the dining centres indicating how many servings of each main course were made daily.	There was no difference in proportion of meat dishes purchased in the control period (87.19%) and in the intervention period (91.58%, *p* > 0.05).	Undesired direction
Information on meat and animal welfare					
Scrim-geour, 2012, [35].	Sample size: N = 363 Age: M = 28 Female: 68% Comparison: Pre-post, Crossover	IG: Information paragraph on the animal welfare implications of eating meat and strategies to eat less.	Whether participants intended to eat less, the same, or more meat in the future was assessed pre- and post-intervention.	Intention to eat less meat was higher at post-intervention (M ≈ 2.3) than at the baseline (M ≈ 2.18, *p* < 0.001, d = 0.65).	Desired direction
Cordts et al., 2014, [36].	Sample size: N = 150 Age: Median: within 40–59 (total study) Female: 55% Comparison: Pre-post	IG: An article on the animal welfare implications of eating meat.	The number of participants intending to eat less meat was assessed pre- and post-intervention by asking whether they would eat less, the same, or more meat in the future.	The percentage of participants intending to eat less meat increased from pre- (15.6%, *N* = 147) to post- intervention (28%, *N* = 150, *p* < 0.001).	Desired direction
Information about the socio-political consequences of meat consumption					
Allen et al., 2002, [42].	Sample Size: IG: *N* = 103, CG: N = 113 Age: Median within: 45–65 Female: 59% Comparison: IG vs CG, CT	IG: Participants were informed that people higher in social dominance orientation (SDO) eat more meat and fewer vegetables, while people lower in social dominance do the opposite. CG: No intervention.	Intended consumption of red and white meat servings in the upcoming 3 days was assessed with a single item directly at post-intervention. Three weeks post-interventions participants reported their consumption of meat servings over the past 7 days.	Intended meat consumption did not differ between IG (High SDO = 1.45, Low SDO = 1.45) and CG (High SDO = 1.56, Low SDO = 1.37). Actual meat consumption remained unchanged in both groups from pre- (IG = 2.88, CG = 2.66) to post-intervention (IG = 2.87, CG = 2.61, *p* > 0.05).	Desired direction (High SDO) Undesired direction (Low SDO)
Cordts et al., 2014, [36].	Sample size: N = 149 Age: Median within: 40–59 (total study) Female: 48% Comparison: Pre-post	IG: An article on the social consequences of eating meat.	The number of participants intending to eat less meat was assessed pre- and post-intervention by asking whether they would eat less, the same, or more meat in the future.	The percentage of participants intending to eat less meat increased from pre- (9%, *N* = 145) to post-intervention (12.1%, *N* = 149, *p* < 0.001).	Desired direction
Information about multiple consequences of eating meat					
Arndt, 2016, study 1, [43].	Sample size: IG: N = 29, CG: *N* = 40 Age: M = 37 (total study) Female: 64% (total study) Comparison: IG vs CG, RCT	IG: Paragraph on the impact of meat consumption of an average American on health, and personal finances, and animal welfare, and the environment, and personal appearance. CG: No intervention.	Intended average daily meat consumption (in servings) was assessed with a single open question directly post-intervention.	Adjusted for baseline meat intake, intended meat consumption did not differ between IG (M = 2.21) and CG (M = 2.63), or among any other study groups (*p* = 0.19).	Desired direction
Arndt, 2016, study 2, [43].	Sample size: IG: *N* = 37, CG: N = 40 Age: 37 (total study) Female: 62% (total study) Comparison: IG vs CG, RCT	IG: Paragraph on the impact of meat consumption on health, and personal finances, and animal welfare, and the environment, also stating that eating less meat can help fulfil one’s altruistic duty. CG: No intervention.	Intended average daily meat consumption (in servings) was assessed with a single open question directly post-intervention.	Adjusted for baseline meat intake, intended meat consumption did not differ between IG (M = 2.24) and CG (M = 2.75), or among any other study groups (*p* = 0.45).	Desired direction
Arndt, 2016, study 2, [43].	Sample size: IG: N = 38, CG: N = 40 Age: 37 (total study) Female: 62% (total study) Comparison: IG vs CG, RCT	IG: Paragraph on the impact of meat consumption on health, and personal finances, and animal welfare, and the environment, also stating that eating less meat could help fulfil one’s personal (egoistic) objectives. CG: No intervention.	Intended average daily meat consumption (in servings) was assessed with a single open question directly post-intervention.	Adjusted for baseline meat intake, intended meat consumption did not differ between IG (M = 1.92) and CG (M = 2.75), or among any other study groups (*p* = 0.45).	Desired direction
Arndt, 2016, study 2, [43].	Sample size: IG: *N* = 30, CG: N = 40 Age: 37 (total study) Female: 62% (total study) Comparison: IG vs CG, RCT	IG: Paragraph on the impact of meat consumption on health, and personal finances, and animal welfare, and the environment. CG: No intervention.	Intended average daily meat consumption (in servings) was assessed with a single open question directly post-intervention.	Adjusted for baseline meat intake, intended meat consumption did not differ between IG (M = 2.57) and CG (M = 2.75), or among any other study groups (*p* = 0.45).	Desired direction
Klöckner et al., 2017, study 1, [44].	Sample size: IG: *N* = 246, CG: *N* = 235 Age: M = 40 (total study) Female: 49% (total study) Comparison: IG vs CG, RCT	IG: Access to one of three subsections of a web-page (selected at random) outlining (a) why to eat less beef, or (b) how to eat less beef, or (c) how to master challenges associated with eating less beef. The webpages included health, and environmental, and social reasons for eating less beef, practical strategies, statements triggering personal values, links to scientific sources, and videos of people’s stories. CG: No intervention.	Change in beef consumption from pre- to 8 weeks post-intervention, was measured with a retrospective food diary.	Adjusted for baseline consumption, there was no difference in the changes in beef consumption between the IG (M = 54.42) and the CG (M = −37.09, simple contrast: *p* = 0.3).	Undesired direction
Klöckner et al., 2017, study 1, [44].	Sample size: IG: *N* = 273, CG: N = 235 Age: M = 40(for total study) Female: 49% (for total study) Comparison: IG vs CG, RCT	IG: Access to a web-page outlining (a) why to eat less beef, and (b) how to eat less beef, and (c) how to master challenges associated with eating less beef. The webpages included health, and environmental, and social reasons for eating less beef, practical strategies, statements triggering personal values, links to scientific sources, and videos of people’s stories. CG: No intervention.	Change in beef consumption from pre- to 8 weeks post-intervention, was measured with a retrospective food diary.	Adjusted for baseline consumption, there was no difference in the changes in beef consumption between the IG (M = 38.17) and the CG (M = −37.09, simple contrast: *p* = 0.79).	Undesired direction
Klöckner et al., 2017, study 2, [44].	Sample size: IG: *N* = 975, CG: *N* = 970 Age: M = 43 (for total study) Female: 47% (for total study) Comparison: IG vs CG, RCT	IG: Access to one of three subsections of a web-page (selected at random) outlining (a) why to eat less beef, or (b) how to eat less beef, or (c) how to master challenges associated with eating less beef. The webpages included health, environmental, and social reasons for eating less beef, practical strategies, statements triggering personal values, links to scientific sources, and videos of people’s stories. CG: No intervention.	Change in beef consumption from pre- to 8 weeks post-intervention, was measured with a retrospective food diary.	Adjusted for baseline consumption, there was no difference in the changes in beef consumption between the IG (M = 28.5) and the CG (M = −66.38, simple contrast: *p* = 0.79).	Undesired direction
Klöckner et al., 2017, study 2, [44].	Sample size: IG: *N* = 974, CG: *N* = 970 Age: M = 43 (for total study) Female: 47% (for total study) Comparison: IG vs CG, RCT	IG: Access to a web-page outlining (a) why to eat less beef, and (b) how to eat less beef, and (c) how to master challenges associated with eating less beef. The webpages included health, environmental, and social reasons for eating less beef, practical strategies, statements triggering personal values, links to scientific sources, and videos of people’s stories. CG: No intervention.	Change in beef consumption from pre- to 8 weeks post-intervention, was measured with a retrospective food diary.	Adjusted for baseline consumption, there was a significant difference in the changes in beef consumption between the IG (M = 13) and the CG (M = −66.38, simple contrast: *p* < 0.001) but these changes went in the undesired direction.	Undesired direction
Berndsen et al., 2005, study 2, [34].	Sample size: IG: *N* = 45, CG: *N* = 47 Age: M = 20.6 Female: 58% Comparison: IG vs. CG, CT	IG: Paragraph on animal welfare, and health, and environmental impact of eating meat. CG: No intervention control.	Three weeks post-intervention, participants reported whether they ate less meat in the past 3 weeks. Directly post-intervention, participants reported if they intended to eat less meat over the upcoming 3 weeks. The scales ranged from 1(fully disagree) to 9 (fully agree).	There was no significant main effect of condition (*p* < 0.10). Interaction and post-hoc tests were not reported.	N/A
Loy et al., 2016, [45].	Sample size: IG: *N* = 28 Age: M = 22 Female: 82% Comparison: Pre-post	IG: A paragraph on the environmental, and ethical, and health, and socio-economic consequences of eating meat and written instructions for mental contrasting and intention implementation.	Meat consumption in g/d was assessed with a 7-day diary the week pre- and the week directly post-intervention.	Meat consumption decreased significantly from pre- to post-intervention (average reduction: 45.2 g/d, *p* < 0.001, d = 1.09).	Desired direction
Loy et al., 2016, [45].	Sample size: IG: N = 30 Age: M = 23 Female: 75% Comparison: Pre-post	IG: A paragraph on the environmental, and ethical, and health, and socio-economic consequences of eating meat.	Meat consumption in g/d was assessed with a 7-day diary the week pre- and the week directly post-intervention.	Meat consumption decreased significantly from pre- to post-intervention (average reduction: 26.4 g/day, *p* = 0.001, d = 0.63).	Desired direction
Marette et al., 2016, [46].	Sample size: 124 (recruited) Age: Median: within 40–49 Female: 54% Comparison: Pre-post	IG: Four paragraphs outlining the health and environmental impact of red meat consumption and the respective benefits of alternative soy-based products.	Before and directly post-intervention, participants conducted a virtual food choice task in which they selected 5 items from either beef or soy burgers.	The selection of beef items declined from pre- (M = 3.52) to post-intervention (M = 2.69, *p* < 0.01).	Desired direction
Leidig, 2012, study 1, [47].	Sample size: IG: *N* = 119 Comparison: Retrospective evaluation	IG: The Meatless Monday campaign toolkit was sent to all healthcare accounts in the US and it was posted on Sodexo’s intranet. The toolkit included information about various benefits of eating less meat, and practical suggestions for implementing a Meatless Monday campaign.	Three months post-intervention, the general managers of Sodexo’s healthcare accounts retrospectively assessed the change in meat purchases on a single scale with 5% increments ranging from 1 (10% + decrease) to 7 (10% + increase), with a score of 4 indicating no changes.	Three months post-intervention 8% of accounts reported increases, 35% reported declines, and the rest reported no changes in meat sales. Using the scale’s mid-points as the average changes in sales, an overall decline was observed M = −0.75%, *p* < 0.001).	Desired direction
Leidig, 2012, study 2, [47].	Sample size: IG: *N* = 126 Comparison: Retrospective evaluation	IG: The Meatless Monday campaign toolkit was posted on Sodexo’s intranet for the corporate and governmental accounts to retrieve. The toolkit included information about various benefits of eating less meat, and practical suggestions for implementing a Meatless Monday campaign.	Three months post-intervention, the general managers of Sodexo’s healthcare accounts retrospectively assessed the change in meat purchases on a single scale with 5% increments ranging from 1 (10% + decrease) to 7 (10% + increase), with a score of 4 indicating no changes.	Three months post-intervention 14% of accounts reported increases, 20% reported declines, and the rest reported no changes in meat sales. There was no overall reduction in sales, when using the scale’s mid-points as average changes (M = − 0.33%, *p* = 0.23).	Desired direction
Tailored information provision					
Arndt, 2016, study 1, [43].	Sample size: IG: N = 37, CG: N = 40 Age: M = 37 (total study) Female: 64% (total study) Comparison: IG vs CG, RCT	IG: Tailored paragraph on how strongly a participant’s personal levels of meat consumption affect their health, or personal finances, or animal welfare, or environment, or personal appearance depending on which consequence participants valued. CG: No intervention.	Intended average daily meat consumption (in servings) was assessed with a single open question directly post-intervention.	Adjusted for baseline meat intake, intended meat consumption did not differ between IG (M = 1.70) and the CG (M = 2.63), or among any other study groups (*p* = 0.19).	Desired direction
Arndt, 2016, study 1, [43].	Sample size: IG: N = 37, CG: N = 40 Age: M = 37 (total study) Female: 64% (total study) Comparison: IG vs CG, RCT	IG: Tailored paragraph on how strongly a participant’s personal levels of meat consumption affect their health, and personal finances, and animal welfare, and the environment, and personal appearance. CG: No intervention.	Intended average daily meat consumption (in servings) was assessed with a single open question directly post-intervention.	Adjusted for baseline meat intake, intended meat consumption did not differ between IG (M = 2.57) and the CG (M = 2.63), or among any other study groups (*p* = 0.19).	Desired direction
Arndt, 2016, study 1, [43].	Sample size: IG: N = 36, CG: N = 40 Age: M = 37 (total study) Female: 64% (total study) Comparison: IG vs CG, RCT	IG: Tailored paragraph on the consequences of an average American’s meat consumption on health, or personal finances, or animal welfare, or the environment, or personal appearance, depending on which consequence participants valued. CG: No intervention.	Intended average daily meat consumption (in servings) was assessed with a single open question directly post-intervention.	Adjusted for baseline meat intake, intended meat consumption did not differ between IG (M = 2.69) and the CG (M = 2.63), or among any other study groups (*p* = 0.19).	Undesired direction
Arndt, 2016, study 2, [43].	Sample size: IG: *N* = 42, CG: N = 40 Age: 37 (total study) Female: 62% (total study) Comparison: IG vs CG, RCT	IG: Tailored paragraph on the consequences of meat consumption on health, or personal finances, or animal welfare, or the environment, depending on which consequence participants valued. The message stated that eating less meat is congruent with being responsible, or adventurous, or logical, or compassionate depending on participants’ self-schema. CG: No intervention.	Intended average daily meat consumption (in servings) was assessed with a single open question directly post-intervention.	Adjusted for baseline meat intake, intended meat consumption did not differ between IG (M = 2.73) and the CG (M = 2.75), or among any other study groups (*p* = 0.45).	Desired direction
Arndt, 2016, study 2, [43].	Sample size: IG: N = 30, CG: N = 40 Age: 37 (total study) Female: 62% (total study) Comparison: IG vs CG, RCT	IG: Tailored paragraph on the consequences of meat consumption on health, and personal finances, and animal welfare, and the environment. The message stated that eating less meat is congruent with being responsible, or adventurous, or logical, or compassionate depending on participants’ self-schema. CG: No intervention.	Intended average daily meat consumption (in servings) was assessed with a single open question directly post-intervention.	Adjusted for baseline meat intake, intended meat consumption did not differ between IG (M = 2.13) and the CG (M = 2.75), or among any other study groups (*p* = 0.45).	Desired direction
Arndt, 2016, study 2, [43].	Sample size: IG: *N* = 33, CG: N = 40 Age: 37 (total study) Female: 62% (total study) Comparison: IG vs CG, RCT	IG: Tailored paragraph on the consequences of meat consumption on health, or personal finances, or animal welfare, or the environment, depending on which consequence participants valued. The message stated that eating less meat can help fulfil one’s altruistic duty. CG: No intervention.	Intended average daily meat consumption (in servings) was assessed with a single open question directly post-intervention.	Adjusted for baseline meat intake, intended meat consumption did not differ between IG (M = 1.97) and the CG (M = 2.75), or among any other study groups (*p* = 0.45).	Desired direction
Arndt, 2016, study 2, [43].	Sample size: IG: N = 29, CG: N = 40 Age: 37 (total study) Female: 62% (total study) Comparison: IG vs CG, RCT	IG: Tailored paragraph on the consequences of meat consumption on health, or personal finances, or animal welfare, or the environment, depending on which consequence participants valued. The message stated that eating less meat can help fulfil one’s personal (egoistic) objectives. CG: No intervention.	Intended average daily meat consumption (in servings) was assessed with a single open question directly post-intervention.	Adjusted for baseline meat intake, intended meat consumption did not differ between IG (M = 1.55) and the CG (M = 2.75), or among any other study groups (*p* = 0.45).	Desired direction
Arndt, 2016, study 2, [43].	Sample size: IG: N = 29, CG: N = 40 Age: 37 (total study) Female: 62% (total study) Comparison: IG vs CG, RCT	IG: Tailored paragraph on the consequences of meat consumption on health, or personal finances, or animal welfare, or the environment, depending on which consequence participants valued.	Intended average daily meat consumption (in servings) was assessed with a single open question directly post-intervention.	Adjusted for baseline meat intake, intended meat consumption did not differ between IG (M = 2.86) and the CG (M = 2.75), or among any other study groups (*p* = 0.45).	Undesired direction
Klöckner et al., 2017, study 1, [44].	Participants had to be adults Sample size: IG: *N* = 275, CG: N = 235 Age: M = 40 (total study) Female: 49% (total study) Comparison: IG vs CG, RCT	IG: Depending on participants stage of change for eating less beef, they were given access to the ‘stage matched’ subsections of a web-page about: (a) why to eat less beef, or (b) how to eat less beef, or (c) how to master challenges associated with eating less beef. The webpages included health, environmental, and social reasons for eating less beef, practical strategies, statements triggering personal values, links to scientific sources, and videos of people’s stories. CG: No intervention.	Change in beef consumption from pre- to 8 weeks post-intervention, was measured with a retrospective food diary.	Adjusted for baseline consumption, there was no difference in the changes in beef consumption between the IG (M = 81.12) and the CG (M = −37.09, simple contrast: *p* = 0.37).	Undesired direction
Klöckner et al., 2017, study 2, [44].	Sample size: IG: *N* = 976, CG: N = 970 Age: M = 43 (for total study) Female: 47% (for total study) Comparison: IG vs CG, RCT	IG: See Klöckner et al. (2017) tailored intervention above. CG: No intervention.	Change in beef consumption from pre- to 8 weeks post-intervention, was measured with a retrospective food diary.	Adjusted for baseline consumption, there was no difference in the changes in beef consumption between the IG (M = 23.79) and the CG (M = −66.38, simple contrast: *p* = 0.65).	Undesired direction
Implicitly highlighting animal suffering					
Tian, 2016, study 1, [49].	Sample size: IG: *N* = 105, CG: *N* = 166 Age: M = 23 (total study) Female: 79% (total study) Comparison: IG vs CG, RCT	IG: Participants viewed a diagram displaying the meat products from various parts of a cow’s image and were asked to write a paragraph describing the figure. (e) CG: No intervention.	Intention to eat beef was measured with 2 items ranging from 1 (low intention to eat beef) to 7 (high intention to eat beef), directly post-intervention	Intended beef consumption did not differ between CG and IG (Mean difference = −0.16, 95%CI = −0.6 to 0.27, *p* = 0.77).	Undesired direction
Tian, 2016, study 1, [49].	Sample size: IG: *N* = 123, CG: N = 166 Age: M = 23 (total study) Female: 79% (total study) Comparison: IG vs CG, RCT	IG: Participants viewed a picture of a cow with a statement that the cow will be sent to another pasture the next day and were asked to write what would happen to the cow. CG: No intervention.	Intention to eat beef was measured with 2 items ranging from 1 (low intention to eat beef) to 7 (high intention to eat beef), directly post-intervention	Intended beef consumption did not differ between CG and IG (mean difference = 0.11, 95%CI = −0.31 to 0.52, *p* = 0.91).	Desired direction
Tian, 2016, study 1, [49].	Sample size: IG: N = 124, CG: N = 166 Age: M = 23 (total study) Female: 79% (total study) Comparison: IG vs CG, RCT	IG: Participants viewed a picture of a cow with a statement that the cow will be sent to the abattoir the next day and were asked to write what would happen to the cow. CG: No intervention.	Intention to eat beef was measured with 2 items ranging from 1 (low intention to eat beef) to 7 (high intention to eat beef), directly post-intervention	Intended beef consumption did not differ between CG and IG (mean difference = 0.32, 95%CI = − 0.09 to 0.73, p = 0.19).	Desired direction
Tian 2016, study 2, [49].	Sample size: IG: *N* = 129, CG: *N* = 120 Age: M = 32 (total study) Female: 67% (total study) Comparison: IG vs CG, RCT	IG: Participants were given a description of a beef dish together with an image of a cow to highlight its animal origin. CG: No intervention.	Intention to eat beef was measured with 2 items ranging from 1 (low intention to eat beef) to 7 (high intention to eat beef), directly post-intervention	Intended beef consumption did not differ between CG and IG (Mean difference: − 0.05, 95%CI = − 0.47 to 0.37, *p* = 0.99).	Undesired direction

(a) Throughout this paper IG and CG respectively refer to Intervention Group and Control Group. (b) Where possible, effect sizes were converted to Cohen’s d using an online tool. (c) Throughout the paper ‘≈’ indicates results that were read from figures or graphs. (d) No inference could be made for the comparison between IG1 and the CG. (e) This intervention was not described in the original paper as being developed to reduce meat consumption, but was included as it highlights the animal origin of meat products

### Interventions’ impact on the demand for meat

#### Individual lifestyle counselling

Six studies (*N* = 2 RCT, *N* = 1 CT, *N* = 3 pre-post) evaluated the effectiveness of six lifestyle counselling interventions to reduce red and/or processed meat consumption [25–30]. All found evidence that lifestyle counselling led to [25, 26] or was associated with reduced meat consumption [27–30]. However, two studies measuring red and processed meat separately, only found significant reductions in the consumption of the latter [28, 29]. Lifestyle counselling interventions were delivered individually by a trained health professional through multiple face-to-face and/or phone sessions and lasted from 6 weeks [28, 29] to 1 year. All counselling interventions additionally comprised written supporting material, with two interventions tailoring this material to individuals [25, 26]. Lifestyle counselling promoted behavioural changes other than meat consumption, including one or more of fruit and vegetable consumption, multivitamin supplement usage, smoking cessation, physical activity, weight loss, and reduction in alcohol consumption. Most counselling interventions (*N* = 5) targeted individuals affected by, or at increased risk of chronic diseases [25, 27–30], and only one such intervention targeted healthy working-class individuals [26].

#### Self-monitoring interventions

Evidence from two RCTs suggested that two self-monitoring interventions reduced red [31] and processed meat consumption [32] during the intervention period and increased intentions to not exceed recommended levels of meat consumption over the week following the intervention. Both interventions lasted 1 week and comprised daily text-messages encouraging participants to self-monitor their red or processed meat consumption with the goal of not exceeding pre-specified recommendations. The intervention focussing on processed meat additionally encouraged participants to think about ‘the regret they could experience’ if they exceeded the recommendations [32].

#### Education on meat consumption and health

Five studies (*N* = 2 RCT, *N* = 1 CT, N = 1 CO, N = 1 Pre-post) evaluated seven interventions providing written information [33–36] or informational videos [37] about the health consequences of eating meat. Of these, five interventions led to [33, 37], or were associated with intended reductions in meat consumption directly post-intervention [35, 36]. Among these studies, one RCT found this effect to be sustained 1 week after the intervention [37]. Conversely, neither a cognitively framed nor an affectively framed message about the health consequences of eating meat were found to be associated with intended reductions in meat consumption directly post-intervention and/or 3 weeks later [34]. Two studies (*N* = 1 RCT, N = 1 CT) assessed the impact of two health focussed educational interventions on actual meat consumption but neither found evidence of an effect [34, 37]. Finally, one controlled trial including elderly people compared the effect of four interventions on the selection of meat in a virtual environment and found that messages on meat consumption and health more effectively reduced the selection of meat products when framed factually (i.e. describing the causal link between meat consumption and its consequences) rather than pre-factually (i.e. outlining hypothetical consequences of hypothetical present meat consumption) [38]. Conversely, messages on meat consumption and well-being more effectively reduced intended meat consumption when framed pre-factually rather than factually [38].

#### Education on meat consumption and the natural environment

Six studies (*N* = 3 RCT, *N* = 1 CO, *N* = 2 Pre-post) evaluated eight interventions providing written information about the environmental consequences of meat consumption [33, 35, 36, 39–41]. Six interventions led to [33, 39, 40] or were associated with increased intentions to eat less meat [35, 36] or with fewer meat products being selected in a simulated food choice experiment [40]. These interventions provided information in the form of paragraphs [35, 39], brief articles [36, 40], or websites [33] and two interventions also outlined practical strategies to aid reductions in meat intake [33, 35]. Conversely, one pre-post study found no evidence to suggest that two informational posters on livestock’s water footprint reduced meat purchases in a university canteen [41].

#### Education on meat consumption and animal welfare

Two studies (*N* = 1 crossover, N = 1 pre-post) evaluated two interventions providing written information about the animal welfare implications of consuming meat [35, 36]. Both were associated with significant reductions in intended meat consumption, and showed more promise than comparable messages on the impact of meat consumption on health or the environment. There was no study assessing the impact of such interventions on actual consumption, purchase, or selection of meat.

#### Education on meat consumption and social issues

Two studies (N = 1 CT, N = 1 pre-post) evaluated two interventions focussing on the social consequences or antecedents of eating meat [36, 42]. Providing information about the association between pursuing high meat diets and holding social dominance values was not found to be associated with reductions in intended consumption directly post-intervention or with actual meat consumption 3 weeks later [42]. Conversely, reading an article on the adverse social consequences of high meat diets was associated with increased intentions to eat less meat, though it offered less promise than similar articles on meat consumption and health, the environment, or animal welfare [36].

#### Education on multiple consequences of meat consumption

Nine studies (*N* = 4 RCT, *N* = 1 CT, *N* = 2 Pre-post, N = 2 retrospective evaluations) assessed 14 interventions providing written information about multiple consequences of meat consumption [34, 43–47]. These interventions provided printed or online information about two or more of health, environmental, animal welfare, social, personal appearance, and economic consequences of eating meat. The impact on actual meat consumption was evaluated in seven interventions: two were associated with lower meat intakes directly and 4 weeks post-intervention in pre-post studies [45], four were not found to effectively reduce meat intakes in RCTs [44], and there was insufficient data to make inferences about the effectiveness of the last intervention [34]. There was no evidence that any of five interventions reduced intended consumption directly post-intervention [43] or 3 weeks later [34], while a pre-post study suggested that providing information about the health and environmental consequences of meat consumption was associated with reduced meat selection in a virtual food choice experiment [46]. Actual food purchases were measured following two interventions providing the Meatless Monday toolkit to the US government/corporate accounts or the US healthcare accounts of a large food service company. Only the latter intervention was associated with significant declines in meat purchases [47]. In summary, four of 14 interventions providing written information about multiple consequences of eating meat were associated with significant reductions in meat consumption, purchases or selection, though none of these interventions was assessed in a RCT.

#### Tailored education

Four RCTs evaluated ten tailored educational interventions, none of which was found to effectively reduce actual [44] or intended meat consumption [43]. Specifically, providing information that was matched to participants’ stage of change (for an account on the stages of change model refer to [48]) for eating less beef was not found to reduce beef consumption in two RCTs, and there was no robust evidence that this tailored intervention outperformed its stage-mismatched equivalent [44]. Two RCTs found no evidence that messages tailored to participants’ most valued consequence of meat consumption, and/or to participants’ self-schema (being responsible, or adventurous, or compassionate, or logical), and/or participants’ personal levels of meat intakes reduced their intended meat consumption [43].

#### Interventions implicitly highlighting animal suffering

Two RCTs found no evidence that any of four interventions implicitly highlighting animal suffering reduced intended meat consumption [49]. All such interventions employed a combination of visual and written material leading recipients to reflect upon the animal suffering involved in the production of meat [49]. For example, participants were shown a picture of a cow accompanied by the statement that the cow will be send to a slaughterhouse, and were asked to think about what would happen to the animal. None of the aforementioned studies assessed actual meat consumption or purchases.

### Qualitative comparative analysis

Fifty-five comparisons were included in QCA. Four configurations of intervention characteristics were consistently associated with reductions in actual or intended meat consumption in real or virtual environments among two or more intervention evaluations (Table 4), while six configurations were consistently not found to be associated with these outcomes (Table 5). QCA supported the findings of the narrative synthesis suggesting that the approaches that were consistently associated with reductions in actual meat consumption were self-monitoring and lifestyle counselling interventions. Non-tailored information provision about the detrimental health or environmental consequences of eating meat was consistently associated with reductions in intended consumption or purchases/selection of meat in virtual environments but was not found to be associated with changes in actual behaviour. Tailored and non-tailored interventions elaborating on several consequences of eating less meat and interventions implicitly highlighting animal suffering were not found to be associated with actual or intended consumption, purchase, or selection of meat in real or virtual environment.

**Table 4:** Configurations of intervention components associated with reductions in meat consumption, purchase, or selection in QCA

Non-tailored environmental information Raw coverage: 25%, Internal consistency: 100%	
Outcome:	(1) Reduction in intended consumption or purchase/selection of meat in virtual environments
In the presence of:	(2) Non-tailored (3) information about environmental issues (4) targeting healthy individuals
In the absence of:	Information about (5) health, (6) socio-economic, (7) animal welfare issues, (8) multiple consequences of eating meat, and (9) implicitly highlighting animal suffering, (10) self-monitoring, (11) goal-setting, and (12) lifestyle counselling
Regardless of:	(13) Provision of practical strategies to eat less meat
Non-tailored health information with practical strategies to eat less meat Raw coverage: 8%, Internal consistency: 100%	
Outcome:	(1) Reduction in intended consumption or purchase/selection of meat in virtual environments
In the presence of:	(2) Non-tailored (3) information about health issues (4) with practical strategies to eat less meat (5) targeting healthy individuals
In the absence of:	Information about (6) environmental, (7) socio-economic, (8) animal welfare issues, (9) multiple consequences of eating meat, and (10) implicitly highlighting animal suffering, (11) self-monitoring, (12) goal-setting, and (13) lifestyle counselling
Self-monitoring and goal-setting interventions Raw coverage: 8%, Internal consistency: 100%	
Outcome:	(1) Reduction in actual meat consumption, purchase, or selection
In the presence of:	(2) Non-tailored (3) self-monitoring and (4) goal-setting interventions (5) targeting healthy individuals
In the absence of:	Information about (6) health (7) environmental, (8) socio-economic, (9) animal welfare issues, (10) multiple consequences of eating meat, and (11) implicitly highlighting animal suffering, (12) practical strategies to eat less meat, and (13) lifestyle counselling
Lifestyle-counselling for people with, or at increased risk of ill-health Raw coverage: 17%, Internal consistency: 100%	
Outcome:	(1) Reduction in actual meat consumption, purchase, or selection
In the presence of:	(2) Tailored (3) lifestyle counselling (4) targeting people with ill health or at increased risk thereof, and including (5) information on health, (6) self-monitoring, (7) goal-setting, and (8) practical strategies to eat less meat
In the absence of:	Information about (9) environmental, (10) animal welfare, (11) socio-economic issues, (12) multiple consequences of eating meat, and (13) implicitly highlighting animal suffering

Configurations of intervention components associated with reductions in meat consumption, purchase, or selection. The overall solution covers 58% of the 24 interventions included in QCA and associated with reductions in meat consumption, purchase, or selection in all comparisons in which these configurations were evaluated. Raw coverage refers to the percentage of interventions associated with reductions in meat consumption, purchase, or selection covered by an intervention configuration. Raw consistency refers to the percentage of interventions within a configuration being associated with the aforementioned outcomes

**Table 5:** Configurations of intervention components not found to be associated with reductions in meat consumption, purchase, or selection in QCA

Tailored information provision Raw coverage: 26%, Internal consistency: 100%	
Outcome:	(1) Reduction in intended consumption or purchase/selection of meat in virtual environments
In the presence of:	(2) Tailored information about (3) one or more of (4) environmental, (5) health, (6) animal welfare, or (7) socio-economic issues, (8) targeting healthy individuals
In the absence of:	(9) Implicitly highlighting animal suffering, (10) self-monitoring, (11) goal-setting, (12) practical strategies to eat less meat, and (13) lifestyle counselling
Information about multiple issues Raw coverage: 19%, Internal consistency: 100%	
Outcome:	(1) Reduction in intended consumption or purchase/selection of meat in virtual environments
In the presence of:	Information about (2) two or more of (3) health, (4) environmental, (5) animal welfare, and (6) socio-economic issues (7) targeting healthy individuals
In the absence of:	(8) Practical strategies to eat less meat, (9) implicitly highlighting animal suffering, (10) self-monitoring, (11) goal-setting, and (12) lifestyle counselling
Regardless of:	(13) Tailoring
Information about multiple issues and practical strategies Raw coverage: 19%, Internal consistency: 100%	
Outcome:	(1) Reduction in actual meat consumption, purchase, or selection
In the presence of:	Information about (2) two or more of (3) health, (4) environmental, and (5) socio-economic issues (6) targeting healthy individuals, and (7) practical strategies to eat less meat
In the absence of:	Information about (8) animal welfare, (9) implicitly highlighting animal suffering, (10) self-monitoring, (11) goal-setting, and (12) lifestyle counselling
Regardless of:	(13) Tailoring
Interventions implicitly highlighting animal suffering Raw coverage: 13%, Internal consistency: 100%	
Outcome:	(1) Reduction in intended consumption or purchase/selection of meat in virtual environments
In the presence of:	(2) Non-tailored interventions (3) implicitly highlighting animal suffering, (4) among healthy individuals
In the absence of:	Information about (5) environmental, (6) health, (7) socio-economic, (8) animal welfare issues, (9) multiple consequences of eating meat, as well as (10) self-monitoring, (11) goal-setting, (12) practical strategies to eat less meat, and (13) lifestyle counselling.
Non-tailored education on the environment, when actual behaviour is the outcome Raw coverage: 6%, Internal consistency: 100%	
Outcome:	(1) Reduction in actual consumption, purchase, or selection of meat
In the presence of:	(2) Non-tailored (3) information about environmental issues (4) targeting healthy individuals
In the absence of:	Information about (5) health, (6) socio-economic, (7) animal welfare, (8) or multiple issues, as well as (9) implicitly highlighting animal suffering, (10) self-monitoring, (11) goal-setting, (12) practical strategies to eat less meat, and (13) lifestyle counselling.
Non-tailored education on health, when actual behaviour is the outcome Raw coverage: 6%, Internal consistency: 100%	
Outcome:	(1) Reduction in actual consumption, purchase, or selection of meat
In the presence of:	(2) Non-tailored (3) information about health issues (4) targeting healthy individuals
In the absence of:	Information about (5) environmental, (6) socio-economic, (7) animal welfare, (8) or multiple issues, as well as (9) implicitly highlighting animal suffering, (10) self-monitoring, (11) goal-setting, (12) practical strategies to eat less meat, and (13) lifestyle counselling.

Configurations of intervention components consistently not found to be associated with reductions in meat consumption, purchase, or selection. The overall solution covers 84% of the 31 interventions included in QCA and not found to be associated with reductions in meat consumption, purchase, or selection in all comparisons in which these configurations were evaluated. Raw coverage refers to the percentage of interventions not found to be associated with reductions in meat consumption, purchase, or selection covered by an intervention configuration. Raw consistency refers to the percentage of interventions within a configuration not found to be associated with the aforementioned outcomes

### Secondary psychosocial outcomes

Self-monitoring interventions led to more favourable instrumental attitudes (beliefs about the impact of eating meat) but not affective attitudes (feeling about the hedonic aspects of eating meat) towards eating less meat [31, 32]. One self-monitoring intervention additionally enhanced perceived behavioural control [31], while there was no evidence that either influenced subjective social norms [31, 32]. In all five interventions evaluating the impact of providing information about the health consequences of eating meat and measuring attitudes, there was evidence that the intervention led to or was associated with more favourable attitudes towards eating less meat [33–35, 37]. Two out of four interventions providing information on the environmental impact of meat consumption led to or were associated with more favourable attitudes towards eating less meat [33, 35, 39]. One non-tailored educational intervention focussing on animal welfare [35] and another focussing on social antecedents of meat consumption were associated with less favourable attitudes towards meat consumption [42]. Conversely, none of the eight tailored educational interventions or five non-tailored interventions focussing on multiple consequences of meat consumption were found to influence attitudes [34, 43].

### Secondary biological outcomes

The only biomarker of health on which studies reported was weight. Of four studies (*N* = 1 CT, *N* = 3 pre-post) evaluating the impact of lifestyle counselling interventions on weight [27–30] only one intervention, which explicitly focussed on weight management, was associated with a significant reduction in BMI in a pre-post comparison [29].

## Discussion

Evidence from experimental intervention studies suggests that some interventions targeting conscious determinants of human behaviour could contribute towards reducing the demand for meat. Six lifestyle counselling interventions designed to reduce red and processed meat consumption led to [25, 26] or were associated with reduced meat consumption [27–30]. Evidence from RCTs suggested that two self-monitoring interventions reduced red [31] or processed meat consumption [32] during the intervention period and enhanced intentions to not exceed recommended levels of consumption. Providing non-tailored information on the health, or environmental, or socio-political, or animal welfare consequences of eating meat was associated with reduced intended consumption and virtual selection of meat in 16 out of 17 interventions [33–37, 39–42]. However, there was no evidence that any of six such interventions influenced actual behaviour [34, 37, 41, 42]. Only four of 14 interventions providing information about multiple consequences of eating meat were associated with reductions in meat consumption, purchases, or selection [34, 43–47]. None of the ten tailored educational interventions or of the four interventions implicitly highlighting animal welfare issues was found to reduce actual [44] or intended meat consumption [43, 49]. Participants’ attitudes towards eating (less) meat generally mirrored their intentions to do so. There was no robust evidence pertaining to any of the other secondary psychosocial outcomes or biomarkers of health risk.

### Strengths and limitations

In this systematic review we employed gold standard methods striving to achieve robust and unbiased results. The scope of our review was kept broad to allow capturing a wide range of intervention approaches, which is important to inform this novel field of research. However, the results should be interpreted in the context of limitations of this review and of the studies included, most of which were of medium or low methodological quality. Having employed extensive methods to identify literature beyond the databases searches, over 40% of the papers included were unpublished records. This potentially prevented publication bias affecting the review unduly, but it also increased the proportion of studies with methodological limitations. Given this field is developing, we elected to include non-randomised studies, which increases the risk of bias in the findings. Additionally, the main outcome measures of this review presented challenges, with self-reported measures of consumption being prone to bias [50], food selection in virtual settings potentially lacking external validity [51, 52], and behavioural intentions being only moderately related to future behaviour [53]. Most studies only measured outcomes during or shortly after the intervention, so conclusions cannot be drawn on lasting effects. Many interventions were either underpowered, making potentially effective interventions difficult to detect, or were evaluated in non-randomised designs, precluding direct causal inference of effectiveness. Future studies should employ well-powered RCTs with longer follow-up to allow for causal inferences to be drawn on the interventions’ effectiveness and to better understand their longer term impact. The studies reviewed included predominantly white and well-educated volunteers, limiting the generalizability of the data to other population groups. Particular caution should be exercised when interpreting results of lifestyle counselling, as our search algorithm might have failed to identify all such interventions. Titles and abstracts of these studies often referred to ‘multiple health behaviours’ as the intervention target, and it is possible that papers specifically mentioning ‘meat consumption’, and therefore identified by our search algorithm, were more likely to be those finding significant results for this outcome. None of the interventions directly targeted gender-related barriers to reduce the demand for meat. Considering the importance of gender as a determinant of meat consumption, future studies should explore whether interventions targeting gender-related barriers can effectively reduce the demand for meat. In the absence of a taxonomy to classify different interventions targeting conscious determinants of human behaviour we grouped interventions according to some of their key behavioural strategies and implementation features. Our synthesis was partly based on an exploratory crisp-set QCA, which represents a sophisticated technique to descriptively identify patterns within data, but not to infer causality of effects. In particular, combinations associated with no evidence of effectiveness should not be thought as ‘ineffective’, as ‘absence of evidence does not equal evidence of absence’ [54]. Similarly, since we did not exclusively include RCTs, combinations associated with effectiveness do not necessarily support an underlying causal mechanism. It must also be considered that QCA did not differentiate between studies of different quality and size. Finally, while the quality assessment tool employed in this review allowed assessing different study designs, caution must be exercised when comparing quality scores between different study designs.

### Findings in the context of existing evidence

The results of this review were consistent with past research on behaviour change interventions targeting other health behaviours. Lifestyle counselling and self-monitoring interventions have previously emerged as promising approaches to change different eating behaviours [55–57]. However, the resources needed to implement lifestyle counselling pose a barrier to its scalability. Moreover, existing evidence on lifestyle counselling targeting the general population rather than people suffering from ill-health is less clear [55], suggesting that our findings on lifestyle counselling may not apply to the general population. Educational interventions providing rational reasons for eating less meat may have underpinned the observed changes in conscious intentions. Nevertheless, unconscious psychosocial and environmental cues that influence behaviour in real-life settings [20, 58] may have prevented these effects from translating into actual behaviour outside experimental settings. Interventions targeting unconscious determinants of human behaviour [20, 58, 59] may therefore play an important role to help overcome this intention-behaviour gap. We plan to review the effectiveness of these approaches elsewhere [60]. The non-significant findings on tailored education are in contrast with the literature highlighting the importance of tailoring information [61, 62]. Nevertheless our findings were based on only two papers that found no evidence of effectiveness for any of the interventions they tested, regardless of tailoring [43, 44]. It is therefore possible, that intervention characteristics other than tailoring or methodological limitations of the aforementioned studies contributed to these non-significant results. However, it is also possible that individuals do not substantially benefit from receiving information about issues that they already value as important consequences of meat consumption, as these arguments might have naturally exhausted their potential for prompting behavioural change. Finally, while providing information was not found to directly influence behaviour, future research should explore whether this approach might contribute towards reducing population-wide demand for meat in other ways. For example, providing information on the benefits of eating less meat might increase the public’s acceptability for more structural interventions to reduce meat consumption.

## Conclusion

This review is the first systematic synthesis of evidence about the effectiveness of interventions targeting conscious determinants of human behaviour to reduce the demand for meat. Some interventions targeting conscious determinants of human behaviour have the potential to reduce the demand for meat. In particular, self-monitoring and individual lifestyle counselling interventions showed promise in reducing actual consumption of meat. Education about health, environmental, socio-political, or animal welfare consequences of eating meat can reduce intended meat consumption and selection of meat in virtual environments, but there was little evidence on whether this approach influenced actual behaviour and the few studies examining this found no evidence that it did. Interventions providing information on several consequences of meat consumption, those implicitly highlighting animal suffering in the context of meat production or consumption, and those providing tailored information offered less promise. While the impact of interventions targeting conscious determinants of human behaviour was modest, if delivered at scale these interventions could contribute towards reducing the demand for meat at population-level.

## Additional file

Additional file 1: **Table S1.** Database search strategy. Algorithm used to search the databases (example for MEDLINE) and list of databases searched. **Table S2.** Searches conducted in publicly accessible online resources. **Table S3.** Interventions’ impact on or association with the demand for meat at the longest follow-up. **Table S4.** Interventions’ impact on or association with attitudes, perceived behavioural control, and subjective social norms of eating meat at both follow-up. **Table S5.** Interventions’ impact on or association with biomarkers of heath risk at both follow-up. (DOCX 61 kb)

## Abbreviations

BMI: Body mass index
CT: Controlled trial
QCA: Qualitative Comparative Analysis
RCT: Randomised controlled trial
