# Supplementary material for: Interventions targeting conscious determinants of human behaviour to reduce the demand for meat: a systematic review with qualitative comparative analysis
Source: Int J Behav Nutr Phys Act. 2018 Oct 19;15:102. doi: 10.1186/s12966-018-0729-6 (PMC6194670; doi:10.1186/s12966-018-0729-6)
Supplement: Supplementary file 1 — Table S1. Database search strategy. Algorithm used to search the databases (example for MEDLINE) and list of databases searched. Table S2. Searches conducted in publicly accessible online resources. Table S3. Interventions’ impact on or association with the demand for meat at the longest follow-up. Table S4. Interventions’ impact on or association with attitudes, perceived behavioural control, and subjective social norms of eating meat at both follow-up. Table S5. Interventions’ impact on or association with biomarkers of heath risk at both follow-up. (DOCX 61 kb) [file 12966_2018_729_MOESM1_ESM.docx]

**Additional file 1**

Table S1 Database search strategy. Algorithm used to search the databases (example for MEDLINE) and list of databases searched.

| Block | Keywords (MEDLINE) | | Hits (MEDLINE) |
| --- | --- | --- | --- |
| 1 | ((meat* or beef or veal or lamb or pork or poultry or chicken or turkey or venison or offal* or sausage* or ham or pastrami or bacon or salami or nugget* or burger* or hamburger* or black pudding* or haggis or plant-based or vegetable-based or vegetar* or vegan* or flexitarian* or ((green or environment* or sustainab*) adj2 (eat or eating or diet*))) adj7 (chang* or increas* or decreas* or improv* or reduc* or low or moderat* or shift* or eliminat* or replac* or substitut* or alternat* or cut* or more or less or free or sustainab* or promot* or encourag* or enhance* or support* or motivat* or achiev*) adj7 (consum* or intak* or eat* or purchas* or choos* or select* or prefer* or demand* or buy* or avoid* or choice* or nudg* or cook* or prepar* or cater* or behav* or inten* or attitud* or social* norm*)).ti,ab. | | 6781 |
| 2 | ((meat* or beef or veal or lamb or pork or poultry or chicken or turkey or venison or offal* or sausage* or ham or pastrami or bacon or salami or nugget* or burger* or hamburger* or black pudding* or haggis or plant-based or vegetable-based or vegetar* or vegan* or flexitarian* or ((green or environment* or sustainab*) adj2 (eat or eating or diet*))) adj7 (consum* or intak* or eat* or purchas* or choos* or select* or prefer* or demand* or buy* or avoid* or choice* or nudg* or cook* or prepar* or cater* or behav* or inten* or attitud* or social* norm*) adj7 (chang* or increas* or decreas* or improv* or reduc* or low or moderat* or shift* or eliminat* or replac* or substitut* or alternat* or cut* or more or less or free or sustainab* or promot* or encourag* or enhance* or support* or motivat* or achiev*)).ti,ab. | | 7008 |
| 3 | ((chang* or increas* or decreas* or improv* or reduc* or low or moderat* or shift* or eliminat* or replac* or substitut* or alternat* or cut* or more or less or free or sustainab* or promot* or encourag* or enhance* or support* or motivat* or achiev*) adj7 (consum* or intak* or eat* or purchas* or choos* or select* or prefer* or demand* or buy* or avoid* or choice* or nudg* or cook* or prepar* or cater* or behav* or inten* or attitud* or social* norm*) adj7 (meat* or beef or veal or lamb or pork or poultry or chicken or turkey or venison or offal* or sausage* or ham or pastrami or bacon or salami or nugget* or burger* or hamburger* or black pudding* or haggis or plant-based or vegetable-based or vegetar* or vegan* or flexitarian* or ((green or environment* or sustainab*) adj2 (eat or eating or diet*))) adj7 (consum* or intak* or eat* or purchas* or choos* or select* or prefer* or demand* or buy* or avoid* or choice* or nudg* or cook* or prepar* or cater* or behav* or inten* or attitud* or social* norm*)).ti,ab. | | 6734 |
| 4 | 1 or 2 or 3 | | 7722 |
| 5 | ((randomized controlled trial or controlled clinical trial).pt. or randomized.ab. or placebo.ab. or drug therapy.fs. or randomly.ab. or trial.ab. or groups.ab.) not (exp animals/ not humans.sh.) | | 3647928 |
| 6 | (((feasability or pilot or demonstration or multicentre* or multicentre* or multi-centre* or multi-center* or preliminary or follow-up or major) adj2 (stud* or project?)) or intervention or preintervention or postintervention or pre-post or (pre adj5 post) or quasiexperiment* or quasi-experiment* or quasirandom* or quasi-random or (before adj10 (after or during)) or ("time series" adj2 interrupt*) or ("time points" adj3 (over or multiple or day? or week? or month?))).mp. not (exp animals/ not humans.sh.) | | 1591076 |
| 7 | 5 or 6 | | 4672088 |
| 8 | 4 and 7 | | 1845 |
| Other databases searched: | | CAB Abstracts (1973 to 2017 Week 33), Embase (1974 to 2017 August 30), PsycINFO (1967 to August Week 3 2017), Science Citation Index (1945-present), MEDLINE (1946-present), and Dissertations & Theses | |

Table S2 Searches conducted in publicly accessible online resources

| Web-page | URL | Date searched | Pathway | Keywords used (if any) | Number of resources screened |
| --- | --- | --- | --- | --- | --- |
| LSE Behaviour Science | <http://blogs.lse.ac.uk/behaviouralscience/posts/> | 09.06.17 | Browsed 'posts' pages | N/A | 3 pages, 29 titles |
|  |  | 09.06.17 | Browsed 'research' pages | N/A | 1 page, 10 titles |
|  |  | 09.06.17 | Keywords in search mask | meat | 1 page, 1 title |
| Behaviour and health Research Unit | <http://www.bhru.iph.cam.ac.uk/?file=index.php> | 09.11.17 | Browsing Research on Diet' | N/A | 1 page, 21 titles |
|  |  | 09.11.17 | Browsing 'Publications' | N/A | 1 page, 70 titles |
| [The Cambridge Green Challenge](https://www.environment.admin.cam.ac.uk/) | [https://www.environment.admin.cam.ac.uk](https://www.environment.admin.cam.ac.uk/) | 09.06.17 | Browsing 'Living Laboratory for Sustainability' | N/A | 1 page, 23 titles |
|  |  | 09.06.17 | Browsing 'Sustainable Food' | N/A | 1 page |
|  |  | 09.06.17 | Browsing 'Case studies'' | N/A | 5 pages, 26 titles |
| Centre for a Liveable Future, John Hopkins | <https://www.jhsph.edu/research/centers-and-institutes/johns-hopkins-center-for-a-livable-future/index.html> | 11.09.17 | Browsing "Research" with keywords ""diet, nutrition and behaviour change" | N/A | 1 page, 86 titles |
| Sustainable Campus - Food (Cornell University) | <http://www.sustainablecampus.cornell.edu/food> | 11.09.17 | Keyword searches | meat | 4 pages, 39 titles |
| Harvard Office for sustainability - Food | <https://green.harvard.edu/topics/food> | 11.09.17 | Keyword searches | meat | 1 page, 33 titles |
| Oxfrod Martin School Future of Food Programmme | <http://www.oxfordmartin.ox.ac.uk/research/programmes/future-food> | 09.06.17 | Browsing 'Publications in future of food' | N/A | 2 pages, 25 |
|  |  |  |  |  |  |
| WWF - Making Food Sustainable | <https://www.wwf.org.uk/what-we-do/area-of-work/making-food-sustainable> | 11.09.17 | Browsed "Food Report" | N/A | 1 page, 10 titles |
|  |  | 11.09.17 | Browsed "Reports" | N/A | 1 page, 31 titles |
|  |  | 11.09.17 | Browsed "Blogs" | N/A | 1 page, 10 titles |
|  |  | 11.09.17 | Browsed "Press Releases" | N/A | 1 page, 192 titles |
|  |  | 11.09.17 | Browsed "News" | N/A | 1 page, 257 titles |
| Meatless Monday | [http://www.meatlessmonday.com](http://www.meatlessmonday.com/) | 09.06.17 | Browsed "Articles" | N/A | 43 pages, 657 titles |
| FAO - Livestock and the environment | <http://www.fao.org/livestock-environment/en/> | 11.09.17 | Browsed "more publications" | N/A | 1 page, 53 titles |
| Better Buying Lab | <http://www.wri.org/our-work/project/better-buying-lab> | 11.09.17 | Browsed "Blog" | N/A | 2 pages, 13 titles |
| European Commission Sustainable Foods | <http://ec.europa.eu/environment/eussd/food.htm> | 11.09.17 | Browsed "Reports and Studies" | N/A | 1 page, 15 titles |
| Environmental Working Group | [http://www.ewg.org](http://www.ewg.org/) | 11.09.17 | Browsed "News" | N/A | 1 page, 8 titles |
|  |  | 11.09.17 | Browsed "Research, Food" | N/A | 1 page, 18 titles |
|  |  | 11.09.17 | Browsed "Key issues, Food, local and sustainable" | N/A | 6 pages, 112 titles |
|  |  | 11.09.17 | Browsed "Key issues, Food, Healthy eating" | N/A | 13 pages, 257 titles |
| Eating Better | [http://www.eating-better.org](http://www.eating-better.org/) | 11.09.17 | Browsed "News and Comment" | N/A | 10 pages, 141 titles |
| Food Climate Research Network | [http://www.fcrn.org.uk](http://www.fcrn.org.uk/) | 09.11.17 | Browsed "Publications" with filters: (1) Livestock and consumption of animal-based foods and (2) Sustainable Healthy Diets and food policy | N/A | 1 page, 23 titles |
| Compassion in World Farming | https://www.ciwf.org.uk | 12.09.17 | Browsed "Research - Food and Human Health" | N/A | 2 pages, 13 titles |
|  |  |  | Browsed "Research - Environment" | N/A | 2 pages, 16 titles |

Table S3 Interventions’ impact on or association with the demand for meat at the longest follow-up.

| **Paper** | **Sample characteristics and comparison** | | **Intervention** | **Outcome** | **Results** |
| --- | --- | --- | --- | --- | --- |
| Information about the health consequences of eating meat | | | | | |
| Fehrenbach, 2015, USA  (37) | | Sample size: IG: N= 59, CG: N=48  Comparison: IG vs CG, RCT | IG: A 4’ video about the health impact of eating meat, highlighting participants’ susceptibility to these outcomes.  CG: No intervention. | Intention to eat less meat in the upcoming 6 months was measured with three 5-points scales, one week post-intervention. | Intention to eat less meat was higher in the IG (M=3.54) than in the CG (M=2.78, *p*<0.001). |
| Fehrenbach, 2015, USA (37) | | Sample size: IG: N=46, CG: N=48  Comparison: IG vs CG, RCT | IG: A 7’ video about the negative health outcomes of eating meat, highlighting participants’ susceptibility to these outcomes, the health benefits of low meat diets, and strategies to eat less meat.  CG: No intervention. | Intention to eat less meat in the upcoming 6 months was measured with three 5-points scales, one week post-intervention. | Intention to eat less meat was higher in the IG (M=3.71) than in the CG (M=2.78, *p*<0.001). |
| Berndsen et al., 2005, Netherlands (Study 1) (34) | | Sample size: IG: N=50, CG: N=38  Comparison: IG vs CG, CT | IG: Cognitively framed paragraph on the health consequences of meat consumption.  CG: No intervention control. | Three weeks post-intervention, participants reported whether they intended to reduce their meat consumption in the future from 1 (fully disagree) to 9 (fully agree). | Intention to eat less meat did not differ between the IG (M=7.50) and the CG (M=7.21, *p*>0.05). |
| Berndsen et al., 2005, Netherlands (Study 1) (34) | | Sample size: IG: N=53, CG: 38  Comparison: IG vs CG,C T | IG1: Affectively framed paragraph on the health consequences of meat consumption.  CG: No intervention control. | Three weeks post-intervention, participants reported whether they intended to reduce their meat consumption in the future from 1 (fully disagree) to 9 (fully agree). | Three weeks post-intervention, intention to eat less meat did not differ between the IG (M=7) and the CG (M=7.21, *p*>0.05). |
| Information about multiple consequences of eating meat | | | | | |
| Berndsen et al., 2005, Netherlands (Study 2) (34) | | Sample size: IG: N=45, CG: N=47  Comparison: IG vs. CG, CT | IG: Paragraph on animal welfare, health, and environmental consequences of eating meat.  CG: No intervention control. | Three weeks post-intervention, participants reported whether they intended to reduce meat consumption in the future on a scale from 1 (fully disagree) to 9 (fully agree). | Intention to eat less meat did not differ between the IG (M=7.20) and the CG (M=7.40, *p*>0.05) |
| Loy et al., 2016, Germany (45) | | Sample size: IG: N=27  Comparison: Pre-post | IG: Paragraph on the environmental, ethical, health, and socio-political consequences of meat consumption including written instructions for mental contrasting and intention implementation. | Meat consumption (in g/d) was assessed using a 7-day diary the week pre- and the fourth week post-intervention | Meat consumption decreased significantly from pre- to post-intervention (average reduction: M=50.3 g/d, *p*<0.001, d=1.18). |
| Loy et al., 2016, Germany (45) | | Sample size: IG: N=28  Comparison: Pre-post | IG: Paragraph on the environmental, ethical, health, and socio-political consequences of meat consumption. | Participants' consumption of meat in g/day way assessed using a 7-day diary was assessed the week pre-and the fourth week post-intervention | Meat consumption decreased significantly from pre- to post-intervention (average reduction: M=27.0g/d, *p*=0.001, d=0.63) |

***Table S4*** Interventions’ impact on or association with attitudes, perceived behavioural control, and subjective social norms at both follow-up.

| **Paper** | | **Comparison** | **Intervention** | **Outcome** | **Results** |
| --- | --- | --- | --- | --- | --- |
| Information about the health consequences of eating meat | | | | | |
| Fehrenbach, 2013, USA (33) | | IG vs CG, RCT | IG: A webpage on the health impact of eating meat, recommending practical strategies to eat less meat.  CG: A webpage about the Rolling Stones. | Attitudes towards cutting out meat once a week were measured directly at post-intervention on four scales from 1 (negative) to 5 (positive). | Attitudes towards cutting out meat once per week were more positive in the IG (M=3.85) than in the CG (M=3.22, *p*<0.001). |
| Fehrenbach, 2015, USA (37) | | IG vs CG, RCT | IG: A 4’ video about the health impact of eating meat, highlighting participants’ susceptibility to these outcomes.  CG: No intervention. | Attitudes towards eating less meat were measured using seven 5-point scales, with higher values indicating more positive attitudes. Attitudes were measured directly post-intervention, and a week post-intervention. | Compared to the CG (M=3.16,) attitudes towards eating less meat were more positive in the IG directly post-intervention (M=3.84, *p*<0.001) and one week post-intervention (CG: M=3.10, IG: M=3.68, *p*<0.001). |
| Fehrenbach, 2015, USA (37) | | IG vs CG, RCT | IG: A 7’ video about the negative health outcomes of eating meat, highlighting participants’ susceptibility to these outcomes, the health benefits of low meat diets, and strategies to eat less meat.  CG: No intervention. | Attitudes towards eating less meat were measured using seven 5-point scales, with higher values indicating more positive attitudes. Attitudes were measured directly post-intervention, and a week post-intervention. | Compared to the CG (M=3.16) attitudes towards eating less meat were more positive in the IG directly post-intervention (M=3.90, *p*>0.001) and one week post-intervention (CG: M=3.10, IG: M=3.78, *p*<0.001). |
| Scrimgeour, 2012, New Zealand (35) | | Pre-post, CO | IG: Information paragraph on the health impact of eating less meat. | Attitudes towards eating meat were measured on 5 scales from 1 (negative) to 5 (positive) at the baseline and directly post-intervention. | Attitudes towards eating meat were more positive at the baseline (M≈3.72) than at post-intervention (M≈3.47, *p*<0.001, d=0.59). |
| Berndsen et al., 2005, Netherlands (Study 1) (34) | | Pre-post | IG: Cognitively framed paragraph on the health impact of eating meat. | Attitudes towards meat were measured with six scales from 1 (negative) to 9 (positive) three weeks post-intervention. | Attitudes towards eating meat were more positive at the baseline (M=6.71) than at post-intervention (M=6.15). No p-value was reported. |
| Berndsen et al., 2005, Netherlands (Study 1) (34) | | Pre-post | IG: Affectively framed paragraph on the health impact of eating meat. | Attitudes towards meat were measured with six scales from 1 (negative) to 9 (positive) three weeks post-intervention. | Attitudes towards meat became significantly less positive from pre- (M= 6.59) to post-intervention (M=5.31, *p*<0.001). |
| Information about the environmental consequences of eating meat | | | | | |
| Fehrenbach, 2013, Germany (33) | | IG vs CG, RCT | IG: A webpage on the environmental impact of eating meat, recommending practical strategies to eat less meat.  CG: Control web-site on the Rolling Stones. | Attitudes towards cutting out meat once a week were measured directly at post-intervention on four scales from 1 (negative) to 5 (positive). | Attitudes towards cutting out meat once per week were more positive in the IG (M=3.68) than in the CG (M=3.22, *p*<0.01). |
| Graham, 2017, New Zealand (39) | | IG vs CG, RCT | IG: A self-transcendent framed paragraph on the livestock related GHG emissions in NZ and the mitigation potential of reduced consumption. | Attitudes towards eating meat weekly were assessed directly post-intervention on six 7-points scales, with higher value indicating more positive attitudes towards having meat. | Attitudes towards eating meat weekly did not differ between the CG (M=4.2) and the IG (M=4.2), or any other study group (*p*>0.05). |
| Graham, 2017, New Zealand (39) | | IG vs CG, RCT | IG: A self-enhancement framed paragraph on the livestock related GHG emissions in NZ and the mitigation potential of reduced consumption. | Attitudes towards eating meat weekly were assessed directly post-intervention on six 7-points scales, with higher value indicating more positive attitudes towards having meat. | Attitudes towards eating meat weekly did not differ between the CG (M=4.2) and the IG (M=4.2), or any other study group (*p*>0.05). |
| Scrimgeour, 2012, New Zealand (35) | | Pre-post, CO | IG: Information paragraph on the environmental impact of eating meat and strategies to reduce consumption. | Attitudes towards eating meat, measured on 5 scales from 1 (negative) to 5 (positive), at the baseline and directly post-intervention. | Attitudes towards eating meat were more positive at the baseline (M≈3.72) than at post intervention (M≈3.48, *p*<0.001, d=0.62) |
| Information on meat consumption and animal welfare | | | | | |
| Scrimgeour, 2012, New Zealand (35) | | Pre-post, CO | IG: Information paragraph on the animal welfare implications of eating meat and strategies to eat less. | Attitudes towards eating meat, measured on 5 scales from 1 (negative) to 5 (positive) at the baseline and directly post-intervention. | Attitudes towards eating meat were more positive at the baseline (M≈3.72) than at post-intervention (M≈3.4, *p*<0.001, d=0.65) |
| Information about the socio-political antecedents and consequences of meat consumption | | | | | |
| Allen et al., 2002, Australia (42) | | IG vs CG, CT | IG: Participants were informed that people higher in social dominance eat more meat and less vegetable, while people lower in social dominance do the opposite.  CG: No intervention. | Attitudes towards red and white meat were assessed directly post-intervention on a scale from 1 (‘bad’) to 7 (‘good’). | Participants with low social dominance orientation (SDO) in the IG had significantly less positive attitudes towards meat (M=4.58) than the CG (M=5.26, *p*<0.01). This effect did not emerge for participants with high SDO. |
| Information about multiple consequences of eating meat | | | | | |
| Arndt (study 1), 2016, USA (43) | | IG vs CG, RCT | IG: Paragraph on the impact of meat consumption of an average American on health, and personal finances, and animal welfare, and the environment, and personal appearance.  CG: No intervention. | Attitudes towards eating meat were assessed directly post-intervention with a scale from 1 (“eating meat is bad") to 7 ("eating meat is good"). | Adjusted for baseline attitudes, post-intervention attitudes towards eating meat did not differ between IG (M=5.38) and CG (M=5.80), or among any other study groups (*p*=0.27). |
| Arndt (study 2), 2016, USA (43) | | IG vs CG, RCT | IG: Paragraph on the impact of meat consumption on health, and personal finances, and animal welfare, and the environment, also stating that eating less meat can help fulfil one's altruistic duty.  CG: No intervention. | Attitudes towards eating meat were assessed directly post-intervention on a scale from 1 (“eating meat is bad") to 7 ("eating meat is good"). | Adjusted for baseline attitudes, post-intervention attitudes towards eating meat did not differ between IG (M=4.97) and CG (M=5.90), or among any other study groups (*p*=0.16). |
| Arndt (study 2), 2016, USA (43) | | IG vs CG, RCT | IG: Paragraph on the impact of meat consumption on health, and personal finances, and animal welfare, and the environment, also stating that eating less meat could help fulfil one’s personal (egoistic) objectives.  CG: No intervention. | Attitudes towards eating meat were assessed directly post-intervention on a scale from 1 (“eating meat is bad") to 7 ("eating meat is good"). | Adjusted for baseline attitudes, post-intervention attitudes towards eating meat did not differ between IG (M=5.16) and CG (M= 5.90), or among any other study groups (*p*=0.16). |
| Arndt (study 2), 2016, USA (43) | | IG vs CG, RCT | IG: Paragraph on the impact of meat consumption on health, and personal finances, and animal welfare, and the environment.  CG: No intervention. | Attitudes towards eating meat were assessed directly post-intervention on a scale from 1 (“eating meat is bad") to 7 ("eating meat is good"). | Adjusted for baseline attitudes, post-intervention attitudes towards eating meat did not differ between IG (M=5.20) and CG (M= 5.90), or among any other study groups (*p*=0.16). |
| Berndsen et al., (study 2), 2005, Netherlands (34) | | IG vs. CG, CT | IG: Paragraph on animal welfare, and health, and environmental impact of eating meat.  CG: No intervention control. | Attitudes towards meat measured with six scales from 1 (negative) to 9 (positive), three weeks post-intervention. | From pre- to post-intervention, attitudes towards meat did not change in a different way between IG and CG (Interaction for time x condition: *p*<0.09). |
| Tailored information provision | | | | | |
| Arndt (study 1), 2016, USA (43) | | IG vs CG, RCT | IG: Tailored paragraph on how strongly a participant’s personal levels of meat consumption affect their health, or personal finances, or animal welfare, or environment, or personal appearance depending on which consequence participants valued.  CG: No intervention. | Attitudes towards eating meat were assessed directly post-intervention on a scale from 1 (“eating meat is bad") to 7 ("eating meat is good"). | Adjusted for baseline attitudes, post-intervention attitudes towards eating meat did not differ between IG (M=5.27), and CG (M= 5.80), or among any other study groups (*p*=0.27). |
| Arndt (study 1), 2016, USA (43) | | IG vs CG, RCT | IG: Tailored paragraph on how strongly a participant’s personal levels of meat consumption affect their health, and personal finances, and animal welfare, and the environment, and personal appearance.  CG: No intervention. | Attitudes towards eating meat were assessed directly post-intervention on a scale from 1 (“eating meat is bad") to 7 ("eating meat is good"). | Adjusted for baseline attitudes, post-intervention attitudes towards eating meat did not differ between IG (M=5.24) and CG (M=5.80), or among any other study groups (*p*=0.27). |
| Arndt (study 1), 2016, USA (43) | | IG vs CG, RCT | IG: Tailored paragraph on the consequences of an average American’s meat consumption on health, or personal finances, or animal welfare, or the environment, or personal appearance, depending on which consequence participants valued.  CG: No intervention. | Attitudes towards eating meat were assessed directly post-intervention on a scale from 1 (“eating meat is bad") to 7 ("eating meat is good"). | Adjusted for baseline attitudes, post-intervention attitudes towards eating meat did not differ between IG (M=5.78) and CG (M=5.80), or among any other study groups (*p*=0.27). |
| Arndt, (study 2), 2016, USA (43) | | IG vs CG, RCT | IG: Tailored paragraph on the consequences of meat consumption on health, or personal finances, or animal welfare, or the environment, depending on which consequence participants valued. The message stated that eating less meat is congruent with being responsible, or adventurous, or logical, or compassionate depending on participants’ self-schema.  CG: No intervention. | Attitudes towards eating meat were assessed directly post-intervention on a scale from 1 (“eating meat is bad") to 7 ("eating meat is good"). | Adjusted for baseline attitudes, post-intervention attitudes towards eating meat did not differ between IG (M=5.47) and CG (M= 5.90), or among any other study groups (*p*=0.16). |
| Arndt, (study 2), 2016, USA (43) | | IG vs CG, RCT | IG: Tailored paragraph on the consequences of meat consumption on health, and personal finances, and animal welfare, and the environment. The message stated that eating less meat is congruent with being responsible, or adventurous, or logical, or compassionate depending on participants’ self-schema.  CG: No intervention. | Attitudes towards eating meat were assessed directly post-intervention on a scale from 1 (“eating meat is bad") to 7 ("eating meat is good"). | Adjusted for baseline attitudes, post-intervention attitudes towards eating meat did not differ between IG (M=5.10) and CG (M=5.90), or among any other study groups (*p*=0.16). |
| Arndt, (study 2), 2016, USA | | IG vs CG, RCT | IG: Tailored paragraph on the consequences of meat consumption on health, or personal finances, or animal welfare, or the environment, depending on which consequence participants valued. The message stated that eating less meat can help fulfil one's altruistic duty.  CG: No intervention. | Attitudes towards eating meat were assessed directly post-intervention on a scale from 1 (“eating meat is bad") to 7 ("eating meat is good"). | Adjusted for baseline attitudes, post-intervention attitudes towards eating meat did not differ between IG (M=5.18) and CG (M=5.90), or among any other study groups (*p*=0.16). |
| Arndt, (study 2), 2016, USA (43) | | IG vs CG, RCT | IG: Tailored paragraph on the consequences of meat consumption on health, or personal finances, or animal welfare, or the environment, depending on which consequence participants valued. The message stated that eating less meat can help fulfil one’s personal (egoistic) objectives.  CG: No intervention. | Attitudes towards eating meat were assessed directly post-intervention on a scale from 1 (“eating meat is bad") to 7 ("eating meat is good"). | Adjusted for baseline attitudes, post-intervention attitudes towards eating meat did not differ between IG (M=5.34) and CG (M= 5.90), or among any other study groups (*p*=0.16). |
| Arndt, (study 2), 2016, USA (43) | | IG vs CG, RCT | IG: Tailored paragraph on the consequences of meat consumption on health, or personal finances, or animal welfare, or the environment, depending on which consequence participants valued. | Attitudes towards eating meat were assessed directly post-intervention on a scale from 1 (“eating meat is bad") to 7 ("eating meat is good"). | Adjusted for baseline attitudes, post-intervention attitudes towards eating meat did not differ between IG (M=5.62) and CG (M= 5.90), or among any other study groups (*p*=0.16) |
| Self-monitoring and goal setting interventions | | | | | |
| Carfora et al., 2017; Italy (32) | IG vs CG, RCT | | IG: Daily text messages for a week, encouraging participants to self-monitor their consumption of processed meat and to 'think about the regret they could experience' if they were to exceed the recommended levels of processed meat consumption (50g/d).  CG: No intervention. | Affective and instrumental attitudes towards eating ≤1 portion of processed meat in the upcoming week were measured with three items on a scale from 1 (negative) to 7 (positive). Perceived behavioural control (PBC) of eating ≤1 portion of processed meat in the upcoming week was measured with seven items on a scale from 1 (low) to 7 (high). Subjective social norms (SSN) of eating ≤1 portion/w of processed meat was measured with three items on a scale from 1 (low) to 7 (high). All outcomes were assessed directly post-intervention. | The IG reported more positive instrumental attitudes (M=6.03) than the CG (M=5.22 *p*<0.004, d=0.63). There were no difference in affective attitudes between the IG (M=4.23) and the CG (M=4.28, *p*>0.05). PBC did not differ between the CG (M=4.54) and the IG (M=4.63, *p*>0.05). SSN did not differ between the CG (M=3.74) and the IG (M=3.93, *p*>0.05). |
| Carfora et al., 2017, Italy (31) | IG vs CG, RCT | | IG: Daily text messages for a week, encouraging participants to self-monitor their consumption of red meat to not exceed a recommended maximum of two medium servings per week.  CG: No intervention. | Affective and instrumental attitudes towards eating <2 medium red meat portions/w in the upcoming week were measured with three items on a scale from 1 (negative) to 7 (positive). PBC of not eating <2 medium red meat portions/w in the upcoming week was measured with seven items on a scale from 1 (low) to 7 (high). SSN of eating <2 medium red meat portions/w was measured with three items on a scale from 1 (low) to 7 (high). All outcomes were assessed directly post-intervention. | The IG reported more positive instrumental attitudes (M= 5.86) than the CG (M=5.09, *p*<0.001, d=0.67). There were no difference in affective attitudes between the IG (M= 4.45) and the CG (M= 4.46, *p*>0.05). PBC was higher in the IG (M=5.23) compared to the CG (M=4.09, *p*<0.05, d=0.29). SSN did not differ significantly between the CG (M=3.98) and the IG (M=4.10, *p*>0.05). |

**Table S5** Interventions’ impact on or association with biomarkers of heath risk at both follow-up.

| **Paper** | **Comparison** | **Intervention** | **Outcome** | **Results** |
| --- | --- | --- | --- | --- |
| Lifestyle counselling | | | | |
| Schiavon et al., 2015, Brazil (27) | IG vs CG, CT | IG: The 12 month intervention targeted consumption of red and processed meat and fruit and vegetables. It provided information bi-weekly phone calls, bimonthly 24-hour dietary recalls followed by researchers’ feedback, and supporting material.  CG: Basic healthy lifestyle guidelines at the baseline and follow-up. | Participants self-reported their weight (in kg) during the phone interviews at post-intervention. | There was no statistical significant difference in post-intervention weight between the control and the intervention group (*p*>0.05) |
| Grimmet et al., 2016, UK (30) | Pre-post | IG: The 12 week intervention targeted consumption of red and processed meat, fruit and vegetables, and physical activity. It comprised 2 weekly telephone calls from the researcher and supporting materials including recipes. The intervention focused on goal- setting, review of goals, self-monitoring, and feedback on performance. | BMI was calculated at the baseline and at the 12 weeks follow-up. | BMI did not differ significantly between the baseline and the follow-up (Mean increase=0.08, *p*=0.60) |
| Hawkes et al., 2009,  Australia (28) | Pre-post | IG: The 6 week intervention targeted consumption of red and processed meat, fruit and vegetable, alcohol, weight management, physical activity, and smoking. It comprised 6 weekly 45’ telephone counselling sessions from a trained health coach, and supporting material. The intervention included lifestyle support, health risks information, behaviour change strategies, self-efficacy, and outcome expectations. | At the baseline and directly post-intervention participants were categorised in underweight (BMI<18.5), healthy weight (BMI=18.5 to 24.9), and overweight/obese (BMI>24.9), based on self-reported data. | There was no change in the proportion of underweight participants between pre- (10%) and post-intervention (10%), there was a decreasing trend in the proportion of healthy weight participants from pre- (25%) to post-intervention (20%), and an increasing trend in the proportion of overweight/obese participants from pre- (65%) to post-intervention (70%). No p-values were reported. |
| Hawkes et al., 2012,  Australia (29) | Pre-post | IG: The 6 week intervention targeted consumption of red and processed meat, fruit and vegetable, alcohol, weight management, physical activity, and smoking. It comprised 6 x 1-hour telephone-coaching sessions with a trained health coach, focussing on motivation, expectations, values, mindfulness, action planning, goal-setting, and self-monitoring, and supporting material. | BMI was assessed using self-reported data at the baseline and directly post-intervention. | There was a significant reduction in BMI from pre- to post-intervention (mean change= -1.4, 95%CI, -2.3 to -0.5, *p*<0.001) |
